# IL-4 Induced Innate CD8^+^ T Cells Control Persistent Viral Infection

**DOI:** 10.1371/journal.ppat.1005193

**Published:** 2015-10-09

**Authors:** Ara Lee, Seung Pyo Park, Chan Hee Park, Byung Hyun Kang, Seong Hoe Park, Sang-Jun Ha, Kyeong Cheon Jung

**Affiliations:** 1 Department of Biochemistry, College of Life Science & Biotechnology, Yonsei University, Seoul, Korea; 2 Transplantation Research Institute, Medical Research Center, Seoul National University College of Medicine, Seoul, Korea; 3 Graduate School of Translational Medicine, Seoul National University College of Medicine, Seoul, Korea; 4 Department of Pathology, Seoul National University College of Medicine, Seoul, Korea; North Carolina State University, UNITED STATES

## Abstract

Memory-like CD8^+^ T cells expressing eomesodermin are a subset of innate T cells initially identified in a number of genetically modified mice, and also exist in wild mice and human. The acquisition of memory phenotype and function by these T cells is dependent on IL–4 produced by PLZF^+^ innate T cells; however, their physiologic function is still not known. Here we found that these IL-4-induced innate CD8^+^ T cells are critical for accelerating the control of chronic virus infection. In CIITA-transgenic mice, which have a substantial population of IL-4-induced innate CD8^+^ T cells, this population facilitated rapid control of viremia and induction of functional anti-viral T-cell responses during infection with chronic form of lymphocytic choriomeningitis virus. Characteristically, anti-viral innate CD8^+^ T cells accumulated sufficiently during early phase of infection. They produced a robust amount of IFN-γ and TNF-α with enhanced expression of a degranulation marker. Furthermore, this finding was confirmed in wild-type mice. Taken together, the results from our study show that innate CD8^+^ T cells works as an early defense mechanism against chronic viral infection.

## Author Summary

Over the course of viral infection there may be a limited time period during which the host system can eliminate the virus. When viruses are not eliminated within this period of time, virus can establish persistent infection. Here, we show that IL-4-induced innate CD8^+^ T cells are able to effectively control chronic virus infection. Innate T cells are heterogeneous population of T cells that acquire effector/memory phenotype as a result of their maturation process in thymus, unlike conventional T cells that differentiate into memory cells after antigen encounter in periphery. Previous data suggest that innate T cells might serve as a first-line of defense against certain bacterial pathogens. IL-4-induced innate CD8^+^ T cells are a unique subset of innate T cells that were recently identified in both mouse and human. We found that IL-4-induced innate CD8^+^ T cells immediately accumulated after viral infection and produced a robust amount of effector cytokines. Thereby, IL-4-induced innate CD8^+^ T cells provide an effective barrier to the establishment of persistent infection via effective virus control during the early phase of viral infection. Collectively our data show that IL-4-induced innate CD8^+^ T cells works as an early defense mechanism against chronic viral infection.

## Introduction

Conventional T cells take on naive phenotypes when they emigrate out from the thymus, whereas innate T cells from the thymus are phenotypically of the effector/memory form [1]. Compared with conventional T cells, these innate T cells, such as natural killer T (NKT) cells, mucosal-associated invariant T (MAIT) cells and H2-M3-specific T cells, are selected by interaction with hematopoietic cells rather than thymic epithelial cells, and their development is dependent on IL–15 and the SAP (SLAM-associated protein) signaling pathway [1]. Moreover, most innate T cells express T cell receptors (TCRs) specific for MHC class Ib molecules [1,2].

Memory-like CD8^+^ T cells expressing eomesodermin (Eomes) are another subset of innate T cells [3]. Although this type of cells is not abundant in wild type C57BL/6 mice, they initially described in Tec-kinase-deficient mice [4,5] and subsequently found in the thymus of a variety of mice in which T-cell-associated genes are deficient [6–13] or CIITA-transgenic (CIITA^Tg^) mice in which MHC class II molecules are expressed in thymocytes [14]. Recently, a substantial number of these innate CD8^+^ T cells was also identified in wild-type BALB/c mice [6] and in human [14]. Eomes^+^ CD8^+^ T cells from both mice and human thymus exhibit immediate effector function upon TCR stimulation [6,14]; however, this type of CD8^+^ T cells has unique characteristics that make them different from MHC class Ib-restricted innate T cells. Firstly, common gamma chain cytokines, particularly IL–4 in this case, drive the expression of Eomes during the intrathymic developmental process [6,14]. Promyelocytic leukemia zinc finger protein (PLZF)^+^ NKT cells are the major source of IL–4 in wild-type BALB/c and Klf2-deficient mice [6], whereas in CIITA^Tg^ mice PLZF^+^ T-T CD4^+^ T cells are responsible for the production of IL–4 [14,15]. In humans, IL–4 would be produced by both PLZF^+^ T-T CD4^+^ T and NKT cells [14]. Secondly, MHC class Ib-restricted innate T cells have a highly restricted TCR repertoire [16], whereas IL-4-induced Eomes^+^ innate CD8^+^ T cells from CIITA^Tg^ mice have a diverse TCR repertoire very much like conventional T cells [14]. This difference in TCR repertoire suggests that they are selected by diverse self-peptides presented by classical MHC class I molecules and raises the possibility that IL-4-induced innate CD8^+^ T cells perform some functions distinct from those of MHC class Ib-restricted innate T cells during a variety of immune responses. However, the biological relevance of IL-4-induced innate CD8^+^ T cells has not been elucidated.

Although CD8^+^ T cells are crucial for the control or elimination of various viral infections, many viruses are able to establish a chronic infection by escaping virus-specific CD8^+^ T cell responses. The functional inactivation of antigen-specific CD8^+^ T cells through the triggering of co-inhibitory receptors such as programmed death–1 (PD–1) and cytotoxic T-lymphocyte antigen–4 (CTLA–4) is currently considered to be a conserved mechanism for not only maintaining viral persistence, but also for limiting immunopathology [17,18]. Moreover, an increase in frequency of virus-specific naïve CD8^+^ T-cell precursors was reported to help control initial viremia but cause differing outcomes with either clearance of wild-type chronic virus or emergence of a T-cell epitope escape mutant virus [19]. However, little is known regarding the impact that the type of CD8^+^ T cells present during infection has on protection against viral persistence.

In the present study, we investigated the *in vivo* role of IL-4-induced innate CD8^+^ T cells in controlling initial viremia using the lymphocytic choriomeningitis virus (LCMV) clone 13 (CL–13) chronic virus infection model. One of the most notable findings from this experiment is that IL-4-induced innate CD8^+^ T cells produce a robust amount of cytokines such as IFN-γ and TNF-α upon LCMV infection, resulting in the efficient control of viruses from the body and providing an effective barrier to the establishment of viral persistence.

## Results

### The anti-viral CD8^+^ T-cell response is enhanced in CIITA^Tg^ mice infected with LCMV CL–13

**Fig 1:**
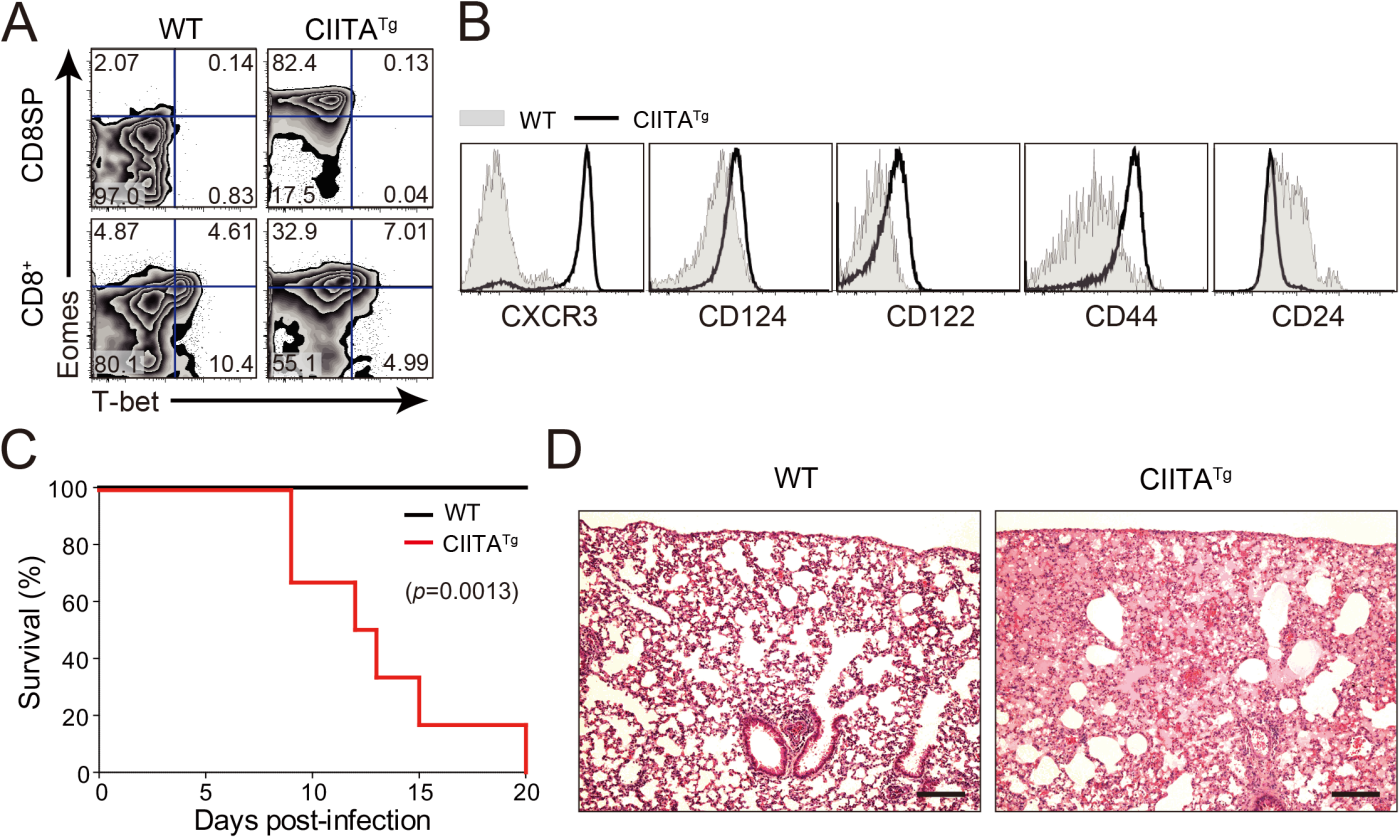
Clone 13 infection caused the immunopathology in CIITA^Tg^ mice with abundant IL-4-induced innate CD8^+^ T cells. (**A**) Cells were isolated from the thymus and spleen of wild-type (WT) and CIITA^Tg^ mice and expression of Eomes and T-bet on CD8 single positive (CD8SP) thymocytes and splenic CD8^+^ T cells were analyzed by flow cytometry. Numbers in the plots indicate the percentages of cells in each quadrant. (**B**) CXCR3, CD124, CD122, CD44, and CD24 expression levels were compared on CD8SP thymocytes of wild-type and CIITA^Tg^ mice. (**C**) Wild-type and CIITA^Tg^ mice were infected with 2 x 10^6^ PFU of LCMV CL–13. Survival of wild-type and CIITA^Tg^ mice were monitored at indicated time points after chronic infection with LCMV CL–13. *P* value = 0.0013, wild-type versus CIITA^Tg^ mice upon conventional dose of LCMV CL–13 infection (log-rank (Mantel-Cox) test). (**D**) Lung pathology in wild-type and CIITA^Tg^ mice at 8 days post-infection (DPI). Data are pooled from two experiments or are representative of at least two experiments (n≥3 per group in each experiment). Original magnification x100, bar = 200 μm.

To explore the *in vivo* function of IL-4-induced Eomes^+^ CD8^+^ T cells, we used CIITA^Tg^ mice in which thymocytes express MHC class II molecules. As reported previously [14], thymus of CIITA^Tg^ mice contain high numbers of Eomes^+^ CD8^+^ T cells, whereas wild-type C57BL/6 mice have only a small number of these cells (Fig 1A). These Eomes^+^ CD8^+^ T cells exhibited a phenotype similar to that of Eomes^+^ memory-like CD8^+^ T cells identified in other types of gene-manipulated mice [3,6] in that they highly express CXCR3, CD124 (IL-4Rα), CD122 (IL-2Rβ) and CD44, and exhibit low expression of CD24 (Fig 1B). We initially infected both CIITA^Tg^ and wild-type mice with a conventional dose (2 x 10^6^ PFU/mouse) of LCMV CL–13 and found that CIITA^Tg^ mice succumbed to early death, whereas wild-type mice did not (Fig 1C). Histopathological analysis of LCMV CL-13-infected CIITA^Tg^ mice showed edematous lungs where most of the alveolar spaces were filled with transudate (Fig 1D), suggesting that the mice died due to immunopathologic tissue damage.

**Fig 2:**
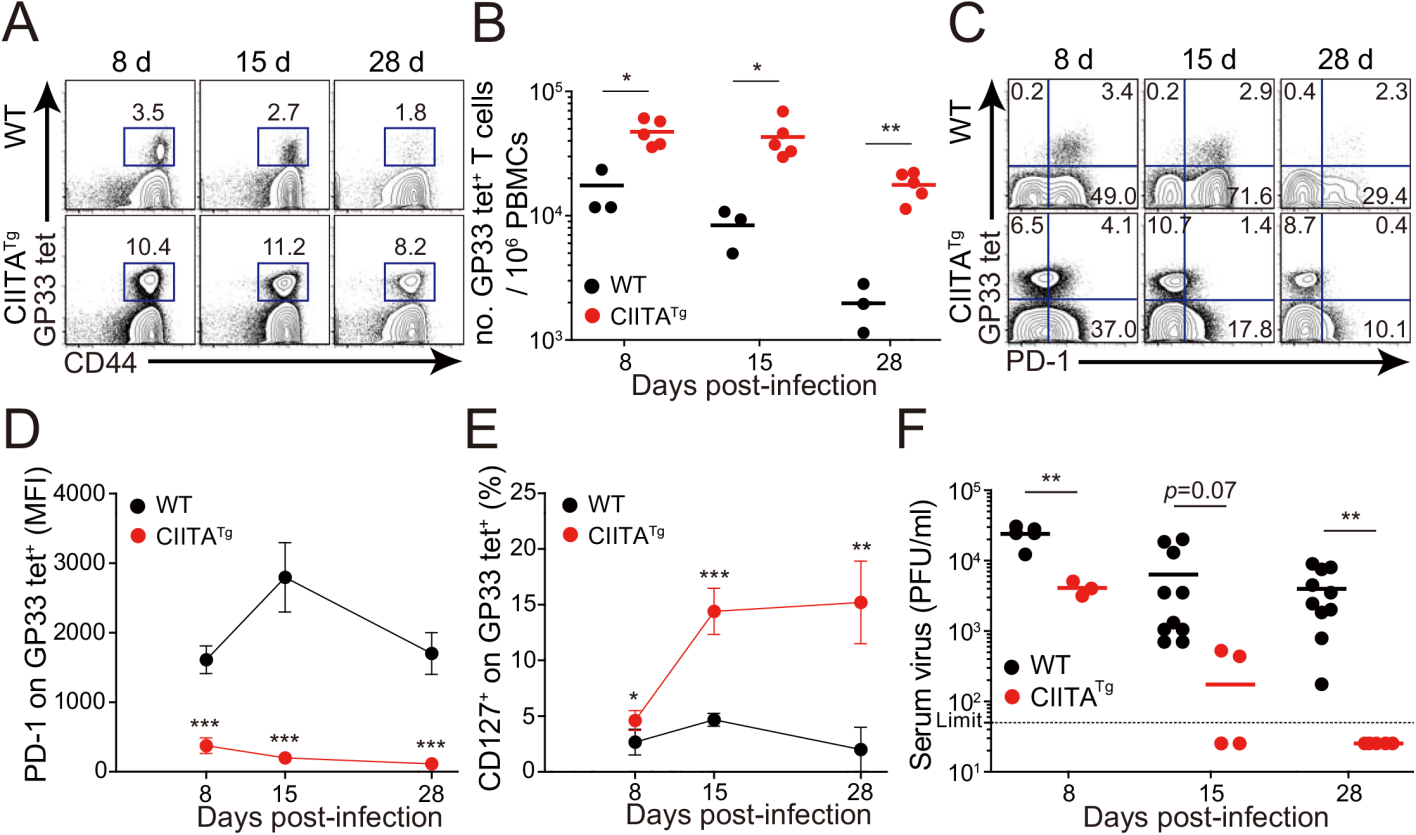
Accelerated viral control in CIITA^Tg^ mice during LCMV clone 13 infection. (**A-E**) Wild-type (WT) and CIITA^Tg^ mice were infected with 5 x 10^5^ PFU of LCMV CL–13 per mouse. Peripheral blood mononuclear cells (PBMCs) were collected at indicated days post infection (DPI). Frequency of GP_33-41_ (GP33) tetramer-positive cells among CD8^+^ T cells were analyzed by flow cytometry (**A**) and the numbers of GP33 tetramer-positive CD8^+^ T cells per 10^6^ PBMCs during the course of LCMV CL–13 infection are represented (**B**). Numbers in plots indicate percentage of the tetramer-positive cells among CD8^+^ T cells. PD–1 (**C and D**) and CD127 (**E**) expression by GP33 tetramer-positive cells after gating on CD8^+^ T cells were also analyzed, and summary showing the PD–1 expression level on GP33 tetramer-positive CD8^+^ T cells is represented by mean fluorescence intensity (MFI) (**D**). The percentage of CD127^+^ cells among GP33 tetramer-positive CD8^+^ T cells is also summarized (**E**). Line graph shows mean ± SD. Data are representatives of four independent experiments (n≥3 per group in each experiment). (**F**) Serum were collected at indicated DPI from the infected wild-type and CIITA^Tg^ mice, and viral titer were checked. Dashed line indicates the virus detection limit. Undetectable samples were given a half of detection limit. Line graph shows mean ± SD. Data of (**A-E**) are representative of three independent experiments, and data of (**F**) is pooled from two independent experiments (n≥3 per group in each experiment).*P<0.05; **P<0.01; ***P<0.001.

We next infected mice with a diverse range of viral doses to determine the minimal infectious dose capable of causing chronic infection in wild-type C57BL/6 mice. We found that inoculation of LCMV CL–13 into wild-type mice established chronic virus infection with sustained expression of PD–1 molecules on CD8^+^ T cells (S1A Fig) and virus persistency in serum (S1B Fig). Based on this, we challenged CIITA^Tg^ and wild-type mice with this dose of LCMV CL–13 for subsequent studies and monitored the frequency of CD8^+^ T cells and viral titer in the blood for 28 DPI. Notably, the frequency and number of endogenous CD8^+^ T cells specific for the LCMV GP_33-41_ epitope (GP33) were greatly enhanced in CIITA^Tg^ mice when compared with those in wild-type mice (Fig 2A and 2B). In addition, sustained expression of PD–1 on GP33-specific CD8^+^ T cells was not induced in CIITA^Tg^ mice upon LCMV CL–13 infection, although wild-type mice exhibited strongly induced PD–1 expression patterns (Fig 2C and 2D). In the CIITA^Tg^ mice, the expression of CD127, which are known to be downregulated on exhausted virus-specific CD8^+^ T cells [19,20] was significantly increased (Fig 2E). This data coincides with a rapid drop in virus titer in blood of CIITA^Tg^ mice, but not in that of wild-type mice (Fig 2F).

**Fig 3:**
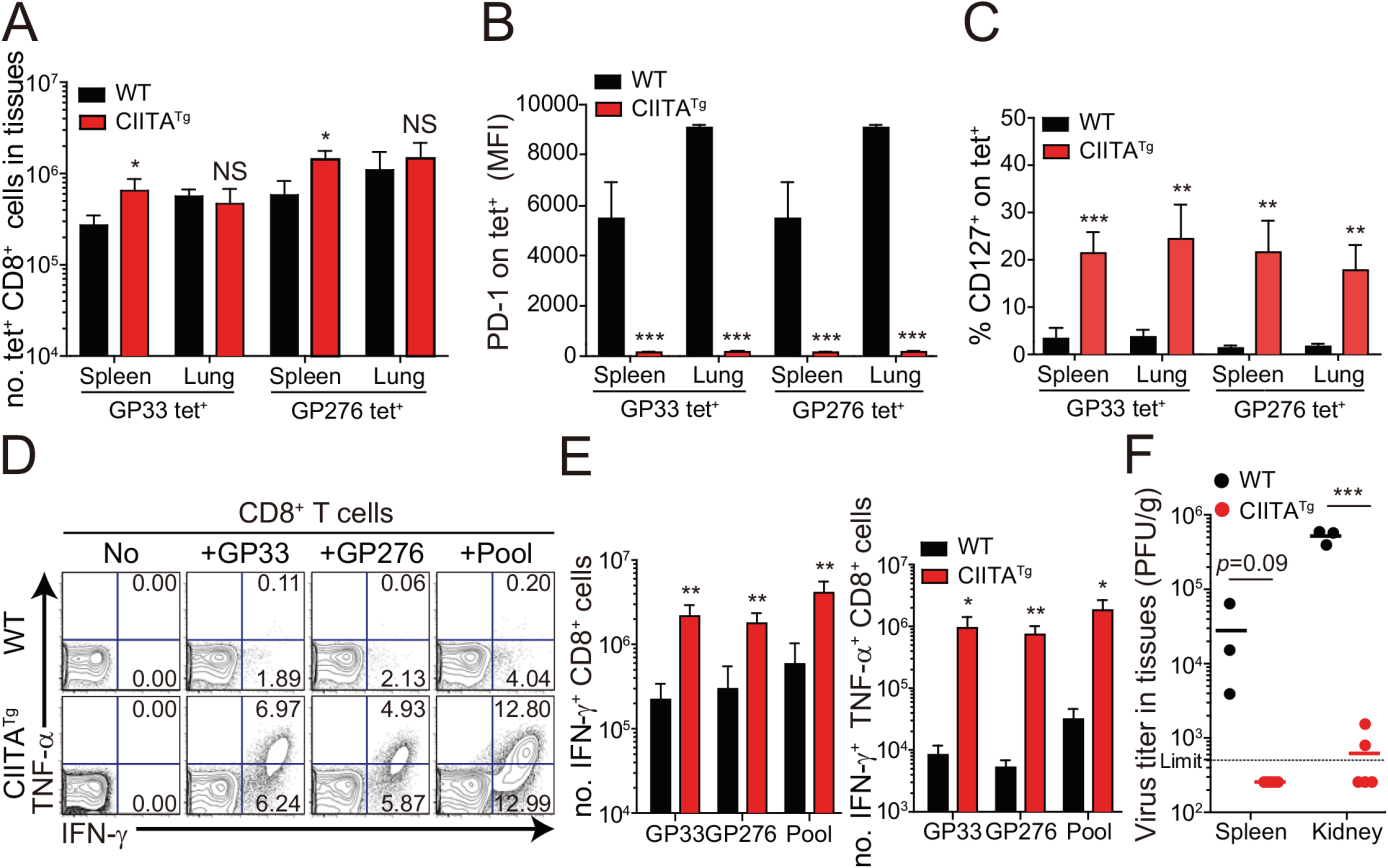
Phenotype and function of virus-specific CD8^+^ T cells in tissues of LCMV infected CIITA^Tg^ mice. (**A-C**) Lymphocytes were isolated from the spleen and lungs of LCMV CL–13 (5 x 10^5^ PFU per mouse) infected wild-type and CIITA^Tg^ mice at 31 DPI and analyzed by flow cytometry. Total numbers of GP33 and GP_276-286_ (GP276) tetramer-positive CD8^+^ T cells (**A**) and PD–1 expression level on tetramer-specific CD8^+^ T cells (**B**) in the spleen and lungs of wild-type and CIITA^Tg^ mice are represented. Percentages of CD127^+^ cells among virus-specific CD8^+^ T cells in the indicated tissues are also summarized in the bar graph (**C**). (**D and E**) Splenocytes were restimulated *in vitro* with GP33, GP276, or LCMV peptide pool (GP33, GP276, GP_118-125_, GP_92-101_, and GP_70-77_) and stained for CD8^+^ T cells. Frequency of IFN-γ- and TNF-α-producing CD8^+^ T cells were analyzed by flow cytometry (**D**), and absolute numbers of CD8^+^ T cells producing IFN-γ and producing both IFN-γ and TNF-α in the spleen are summarized, respectively (**E**). Numbers in the plots indicate the percentage of TNF-α^+^ and TNF-α^-^ CD8^+^ T cells producing IFN-γ, respectively. (**F**) Viral titers were checked in the spleen and kidney extracted from LCMV CL-13-infected mice at 31 DPI. Dashed line indicates the virus detection limit. Undetectable samples were given a half of detection limit. Bar graphs show mean + SD. Data are representative of three independent experiments (n≥3 per group in each experiment). NS, not significant; *P<0.05; **P<0.01; ***P<0.001.

We next asked whether the enhanced CD8^+^ T-cell response was also present in peripheral tissues, particularly during the late phase of viral infection. To this end, we dissected the CD8^+^ T-cell response with respect to their number and cytokine responses in the spleen and lungs at 31 DPI. In this experiment, endogenous CD8^+^ T cells specific for the LCMV GP_276-286_ epitope (GP276) as well as for LCMV GP33 were analyzed phenotypically and functionally. As expected, the CIITA^Tg^ mice contained higher numbers of GP33- or GP276-specific CD8^+^ T cells in the spleen (Fig 3A) and these cells exhibited much lower levels of PD–1 expression on their surface (Fig 3B) compared with wild-type mice. As was the case in peripheral blood, a substantial population of CD127^hi^ virus-specific memory CD8^+^ T cells was detected in the spleen and lung of CIITA^Tg^ mice (Fig 3C). A particularly important point in this experiment is the fact that CD8^+^ T cells from CIITA^Tg^ mice showed very strong cytokine responses compared with T cells from wild-type mice, in this case IFN-γ and TNF-α release upon *ex vivo* restimulation with GP33, GP276, or pooled peptides (Fig 3D and 3E **).** Like that of peripheral blood, this enhanced function of virus-specific CD8^+^ T cells was associated with decreased viral titers in the spleen and especially in the kidney, which is well known to be a life-long reservoir of chronic LCMV, of CIITA^Tg^ mice compared with wild-type mice (Fig 3F **).** Taken together with data from peripheral blood, this strongly suggests that enhanced virus-specific CD8^+^ T-cell responses both quantitatively and qualitatively contribute to the accelerated control of viremia in CIITA^Tg^ mice.

### Mice deficient for IL-4-induced innate CD8^+^ T cells fail to control LCMV CL–13 infection

**Fig 4:**
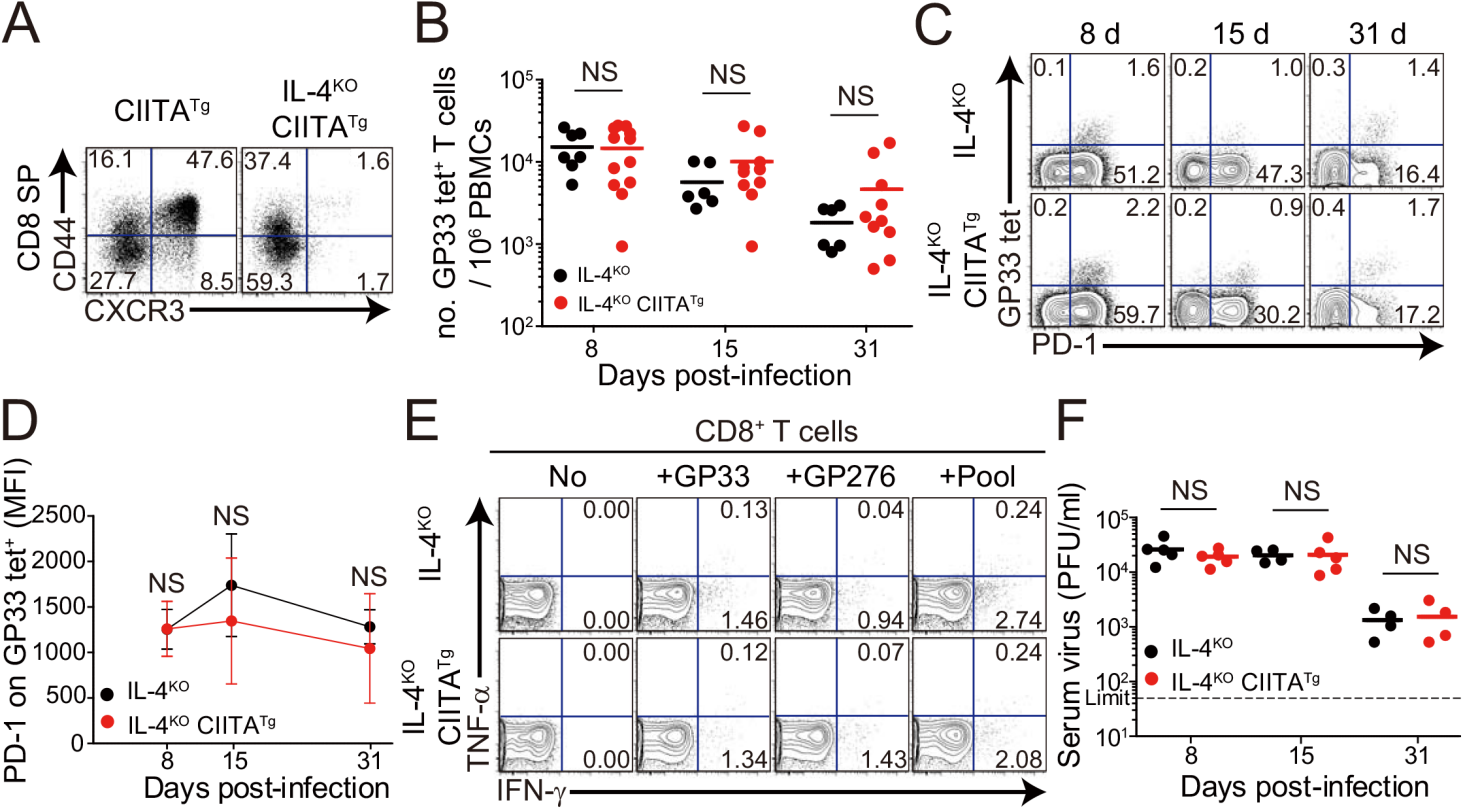
CD8^+^ T cells responses in IL-4-deficient CIITA^Tg^ and control mice during LCMV clone 13 infection. **(A)** Cells were isolated from the thymus of CIITA^Tg^ and IL-4^KO^CIITA^Tg^ mice and expression of CXCR3 and CD44 on CD8 single positive (CD8SP) thymocytes were analyzed by flow cytometry. Numbers in the plots indicate the percentages of cells in each quadrant. (**B-F**) IL-4^KO^ and IL-4^KO^CIITA^Tg^ mice were infected with 5 x 10^5^ PFU of LCMV CL–13 per mouse. PBMCs were collected at the indicated DPI for analysis. The numbers of GP33 tetramer-positive CD8^+^ T cells per 10^6^ PBMCs (**B**) and frequency of GP33 tetramer-specific CD8^+^ T cells and their PD–1 expression among CD8^+^ T cells (**C**) were assessed during the course of LCMV CL–13 infection by flow cytometry. Numbers in the plots indicate percentage of PD–1^+^ or PD–1^-^ GP33 tetramer-positive cells among CD8^+^ T cells. PD–1 expression level on GP33 tetramer-positive CD8^+^ T cells in PBMCs represented by MFI value (**D**). (**E**) Lymphocytes isolated from the spleen of mice at 31 DPI were restimulated *in vitro* with GP33, GP276, or LCMV peptide pool for CD8^+^ T cells. Frequency of IFN-γ- and TNF-α-producing CD8^+^ T cells were analyzed by flow cytometry. (**F**) Serum viral titer in IL-4^KO^ and IL-4^KO^CIITA^Tg^ mice at the indicated DPI. Dashed line indicates the virus detection limit. Line graph shows mean ± SD. Data of (**B-D**) are data pooled from two independent experiments. Data of (**E and F**) are representative of four independent experiments (n≥3 per group in each experiment). NS, not significant.

The development of IL-4-induced innate CD8^+^ T cells is dependent on PLZF^+^ T cells, such as PLZF^+^ innate CD4^+^ T cells [14] and NKT cells, as a source of IL–4 [6]. Consistent with the previous report [14], the frequency of innate CD8^+^ T cells co-expressing CD44 and CXCR3 in the thymus was significantly lower in CIITA^Tg^IL-4^KO^ mice than in CIITA^Tg^ mice (Fig 4A). Thus, to determine whether the enhanced anti-viral CD8^+^ T-cell response in CIITA^Tg^ mice is dependent on IL-4-induced innate CD8^+^ T cells, we used CIITA^Tg^IL-4^KO^ mice. When these mice were infected with CL–13 and virus-specific CD8^+^ T-cell numbers in peripheral blood were monitored until 31 DPI, the overall magnitude of these cells in CIITA^Tg^IL-4^KO^ mice was found to be as low as that in control IL-4^KO^ mice infected with CL–13 (Fig 4B). Functionally, virus-specific CD8^+^ T cells in both CIITA^Tg^IL-4^KO^ and IL-4^KO^ mice exhibited an exhausted phenotype in terms of sustained PD–1 expression (Fig 4C and 4D). In addition, the robust cytokine production exhibited in cells from CL-13-infected CIITA^Tg^ mice upon *ex vivo* restimulation with epitope peptides was not present in CIITA^Tg^IL-4^KO^ mice (Fig 4E). This defect in CD8^+^ T-cell function suggests a failure of virus control in CIITA^Tg^IL-4^KO^ mice. Indeed, virus was not cleared in these mice (Fig 4F). Thus, these results imply that the enhanced CD8^+^ T-cell response in CIITA^Tg^ mice infected with CL–13 is dependent on the existence of IL-4-induced innate CD8^+^ T cells.

It is possible that IL–4 deficiency probably has effects not only on the loss of the IL-4-induced innate CD8^+^ T cells but also on the development of antibody responses, an important component in determining the outcome of virus infection. To exclude the possibility, we examined cytotoxic T lymphocyte (CTL) and antibody response between wild-type and IL-4^KO^ mice during the course of CL–13 infection. The number of virus-specific CD8^+^ T cells and their PD–1 expression were not different between wild-type and IL-4^KO^ mice (S2A and S2B Fig). When serum level of LCMV-specific IgG was measured, the level was also similar between these mice (S2C Fig). Accordingly, absolute numbers of follicular helper T (T_FH_) cells and germinal center (GC) B cells was not different between IL-4^KO^ and wild-type mice (S2D and S2E Fig), which both could not control viruses for a certain period (S2F and S2G Fig). These data suggest that IL–4 itself does not affect the control of chronic viruses.

### IL-4-induced innate CD8^+^ T cells mediate early control of CL–13 infection

**Fig 5:**
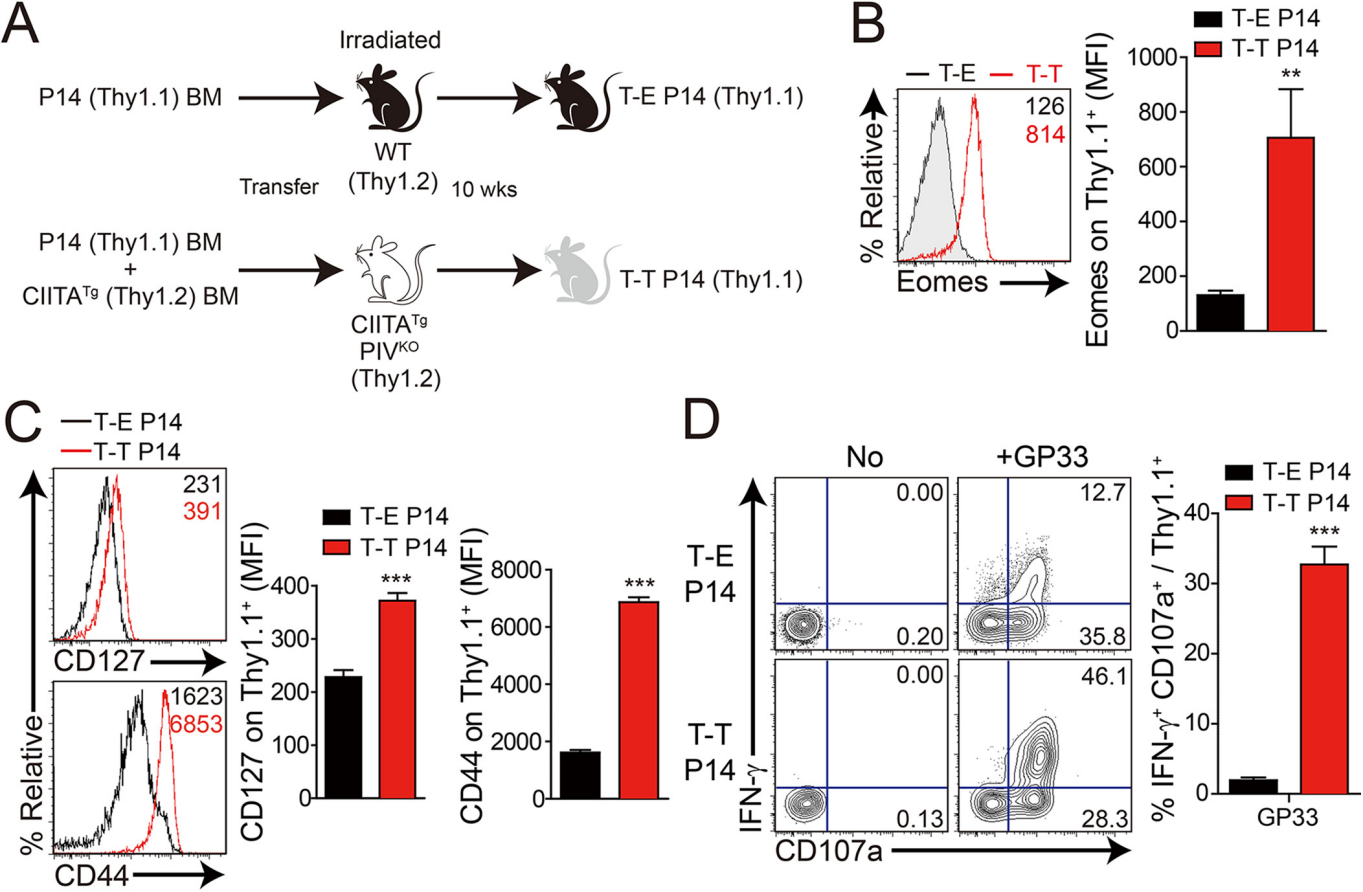
Generation and characterization of virus-specific IL–4 induced innate CD8^+^ T cells. (**A**) Wild-type (WT) and CIITA^Tg^PIV^KO^ host mice (Thy1.2^+^) were sublethally irradiated. Irradiated wild-type and CIITA^Tg^PIV^KO^ mice were reconstituted for 10 weeks with P14 (Thy1.1^+^) BM (T-E P14) and a 1:1 mixture of P14 (Thy1.1^+^) and CIITA^Tg^ (Thy1.2^+^) BM (T-T P14), respectively. (**B and C**) Lymphocytes were isolated from the spleen of T-E and T-T P14 mice. Eomes expression on P14 cells were analyzed by flow cytometry (**B**). Numbers in the plots indicate MFI values of Eomes staining on T-E and T-T P14 cells. MFI values are summarized in the bar graphs. CD127 and CD44 expression level on P14 cells of the spleen in T-E and T-T P14 mice were also analyzed (**C**). Numbers in the plots indicate CD127 and CD44 MFI value on T-E and T-T P14 cells. MFI values of CD127 and CD44 are also summarized in the bar graph. (**D**) Splenocytes from the BM chimeric mice were stimulated *in vitro* with GP33 for P14 cells for 5 hours, and stained with anti-Thy1.1, CD107a, and IFN-γ. Numbers in quadrants indicate the percentage of CD107a^+^ IFN-γ-producing or nonproducing P14 cells. Data were representative from more than two independent experiments and summarized data are shown; bars indicate the mean + SD. **P<0.01; ***P<0.001.

Next, to confirm the anti-viral function of IL-4-induced innate CD8^+^ T cells, we generated these cells in P14 TCR transgenic mice, which express a TCR recognizing LCMV GP33 peptide presented by H-2D^b^ molecules. To produce P14 Eomes^+^ CD8^+^ T cells, we injected a mixture of BM cells isolated from CIITA^Tg^ and Thy1.1 P14 mice into irradiated CIITA^Tg^PIV^KO^ mice (Fig 5A, T-T P14). In CIITA promoter type IV null (PIV^KO^) mice, MHC class II molecules are not expressed only on cortical thymic epithelial cells [21], therefore most of CD4^+^ T cells are selected by MHC class II^+^ thymocyte-thymocyte interaction pathway in CIITA^Tg^PIV^KO^ mice [15]. As a result, CIITA^Tg^PIV^KO^ mice generate much higher number of PLZF^+^ CD4^+^ T cells as compared to CIITA^Tg^ mice, so that these mice are able to get much higher amount of IL–4 in thymic environment. As expected, this mixed chimerism allowed most of the P14 CD8^+^ T cells to express Eomes (Fig 5B). Consistent with a previous report [14], Eomes^+^ P14 cells developed with a CD127^hi^CD44^hi^ memory phenotype (Fig 5C). In contrast, wild-type C57BL/6 mice that received Thy1.1 P14 BM cells contained only Eomes^-^ naïve P14 cells (Fig 5A–5C, T-E P14). Eomes^+^ P14 cells also exhibited enhanced ability for effector cytokine production and cytotoxicity compared with the Eomes^-^ counterpart: a higher fraction of Eomes^+^ cells produced IFN-γ and expressed a marker of degranulation, CD107a, upon *in vitro* stimulation with GP33 peptide (Fig 5D).

**Fig 6:**
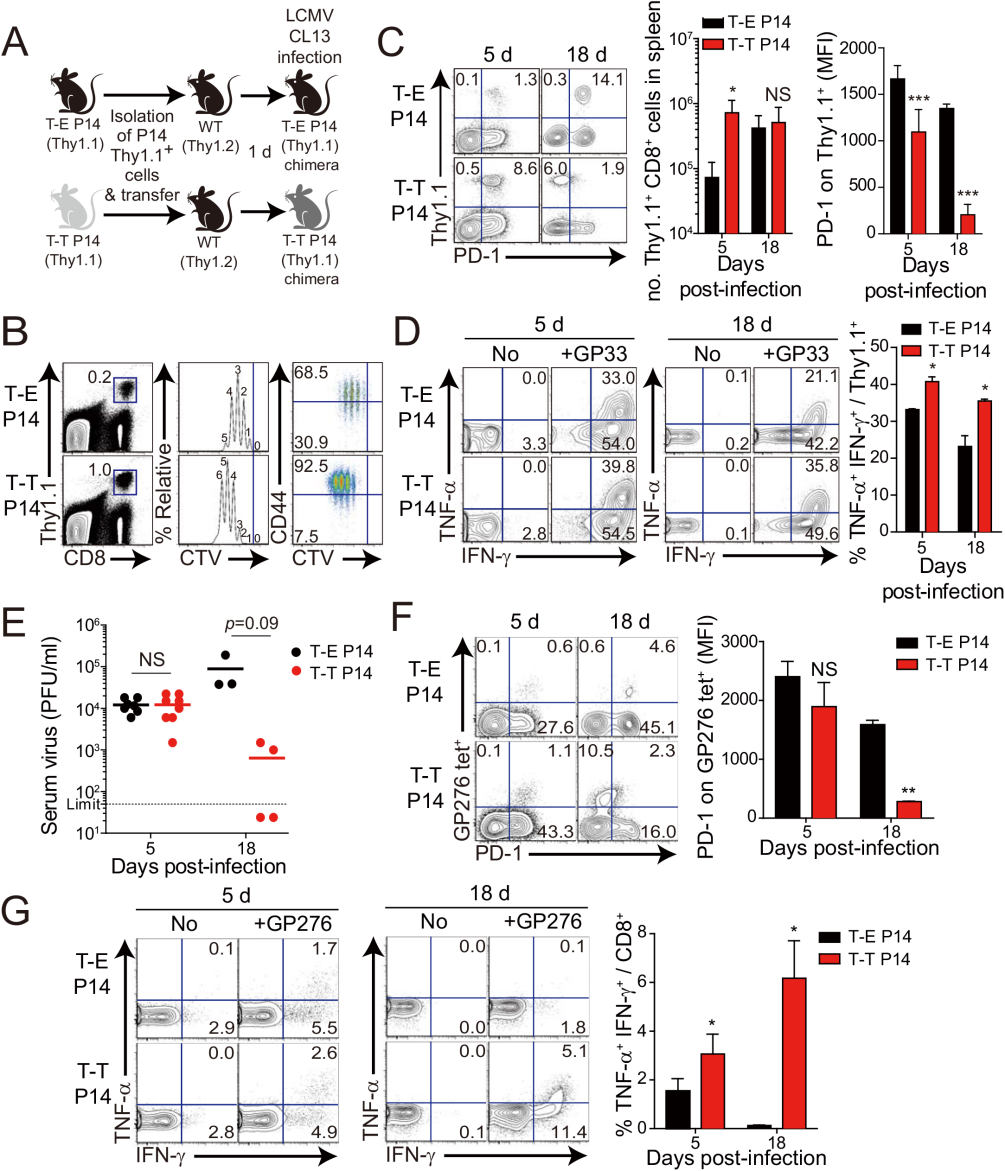
Control of chronic LCMV infection by innate CD8^+^ T cells. (**A**) T-E and T-T P14 cells were isolated from the spleens of each BM chimera as shown in Fig 5 and transferred into Thy1.2^+^ congenic C57BL/6 mice. One day after transfer, the mice were infected with 5 x 10^5^ PFU of LCMV CL–13 per mouse. **(B-D)** Thy1.1^+^ CD8^+^ donor P14 cells were gated for following analysis. **(B)** CellTrace Violet (CTV)-labelled donor T-E and T-T P14 cells were analyzed at 2.5 DPI by flow cytometry. Frequency of Thy1.1^+^ CD8^+^ P14 cells among splenocytes are depicted. The gated donor Thy1.1^+^ CD8^+^ P14 cells are examined for CTV dilution along with CD44 expression. Division time and frequency of CD44^hi^ cell population are indicated in histogram plot. (**C**) Lymphocytes were isolated from the spleens of T-E and T-T P14 chimeric mice at 5 and 18 DPI and analyzed by flow cytometry. The number of Thy1.1^+^ P14 cells in the spleen and PD–1 expression among CD8^+^ T cells were analyzed at indicated DPI. Numbers in the plots indicate PD–1^+^ or PD–1^-^ Thy1.1^+^ transferred cells. PD–1 expression level (MFI) on Thy1.1^+^ transferred cells is summarized in the graph. (**D**) Splenocytes of T-E and T-T P14 chimeric mice were restimulated *in vitro* with GP33 for P14 cells, and frequency of IFN-γ- and TNF-α-producing Thy1.1^+^ transferred cells were analyzed. Numbers in the plots indicate the percentage of TNF-α^+^ and TNF-α^-^ CD8^+^ T cells producing IFN-γ, respectively. Frequency of Thy1.1^+^ transferred cells producing both IFN-γ and TNF-α was summarized in the graph. (**E**) Serum viral titers were checked in T-E and T-T P14 chimeras at the indicated DPI. Dashed line indicates the virus detection limit. Undetectable samples were given a half of detection limit. **(F-G)** CD8^+^ T cells were gated for following analysis. **(F)** PD–1 expression on GP276 tetramer-positive CD8^+^ T cells was analyzed at indicated DPI. Numbers in the plots indicate PD–1^+^ or PD–1^-^ GP276 tetramer-positive cells among CD8^+^ T cells. PD–1 expression level (MFI) on GP276 tetramer-positive CD8^+^ T cells is summarized in the graph. **(G)** Splenocytes of T-E and T-T P14 chimeric mice were restimulated *in vitro* with GP276 peptide for endogenous CD8^+^ T cells, and frequency of IFN-γ- and TNF-α-producing CD8^+^ T cells was analyzed. Numbers in the plots indicate the percentage of TNF-α^+^ and TNF-α^-^ CD8^+^ T cells producing IFN-γ, respectively. Frequency of CD8^+^ T cells producing both IFN-γ and TNF-α was summarized in the graph. Bar graphs show mean + SD. Data are representative of more than two independent experiments (n≥3 per group in each experiment). NS, not significant; *P<0.05; **P<0.01; ***P<0.001.

After generation of virus-specific IL-4-induced innate CD8^+^ T cells via mixed BM chimerism, we evaluated the anti-viral function of these innate CD8^+^ T cells *in vivo*. For this, we transferred Eomes^+^ or Eomes^-^ P14 cells into congenic hosts, which were then infected with CL–13 (Fig 6A). This strategy allowed us to track the response of IL-4-induced and Eomes^-^ conventional CD8^+^ T cells after CL–13 infection and to judge their individual contributions to the protection against viral persistence. To examine the proliferation capability of Eomes^+^ or Eomes^-^ P14 cells after CL–13 infection, each of the cells were labelled and transferred into naïve congenic mice. After 2.5 DPI, Eomes^+^ P14 cells showed a more division and a higher expression of CD44 compared to Eomes^-^ P14 cells (Fig 6B). In the spleen, a 10-fold higher number of P14 cells accumulated at an early time point (5 DPI) in the mice that received Eomes^+^ P14 cells than in those that received Eomes^-^ cells, although these cells were detected with similar abundance at a later time point (18 DPI) (Fig 6C). In the mice that received Eomes^+^ P14 cells, PD–1 was only transiently expressed on P14 cells at 5 DPI and was then later downregulated (Fig 6C). In contrast, the high level of PD–1 expression was sustained on Eomes^-^ P14 cells. Functionally, P14 cells from mice received Eomes^+^ P14 cells were superior to those from recipients of Eomes^-^ cell, with a higher fraction of these cells capable of producing both IFN-γ and TNF-α (Fig 6D). To test whether the increased CD8^+^ T-cell activity corresponded to better viral control we measured viral titer in the serum. Indeed, Eomes^+^ P14 cells also had an effect on reducing viral load during CL–13 infection (Fig 6E). These results indicate that Eomes^+^ P14 cells provide superior support for anti-viral activity compared to Eomes^-^ P14 cells.

Interestingly, most of the responding CD8^+^ T cells of recipients were PD-1-negative in the mice that received Eomes^+^ P14 cells (Fig 6C) compared to those that received Eomes^-^ P14 cells at 18 DPI. Thus, we examined the PD–1 expression on endogenous virus-specific CD8^+^ T cells, which are initially Eomes-negative before infection, and their cytokine production. PD–1 expression on endogenous GP276 tetramer^+^ CD8^+^ T cells was comparable to the donor Eomes^+^ P14 cells (Fig 6F). In addition, endogenous virus-specific CD8^+^ T cells in the mice that received Eomes^+^ P14 cells were not exhausted (Fig 6G). These data suggest that PD–1 expression in both donor Eomes^+^ P14 cells and endogenous virus-specific CD8^+^ T cells during the course of CL–13 infection presumably depend on antigen levels rather than intrinsic property of the Eomes^+^ or Eomes^-^ responding cells.

**Fig 7:**
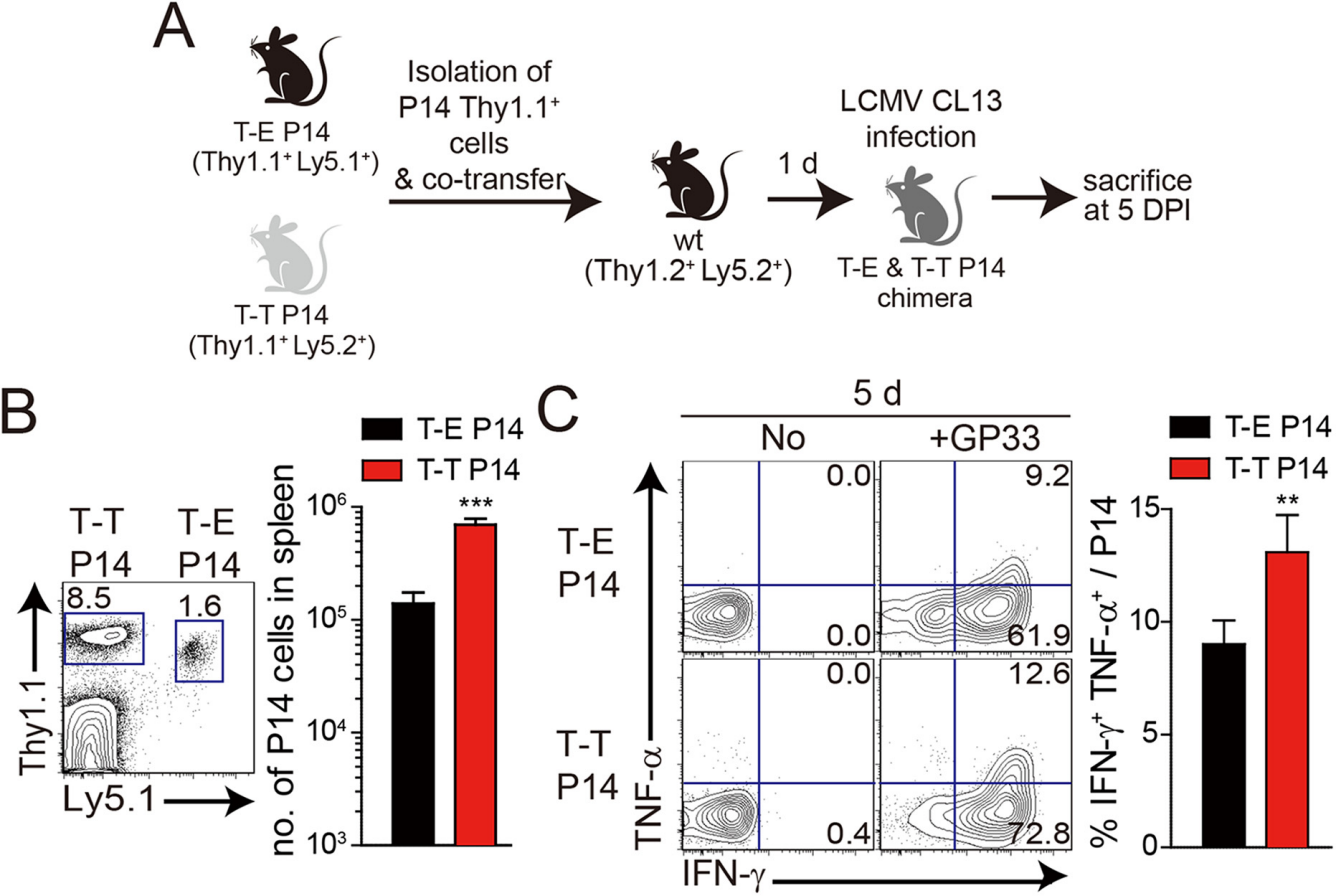
Co-adoptive transfers of T-E and T-T P14 cells. **(A)** T-E P14 (Thy1.1^+^Ly5.1^+^) and T-T P14 (Thy1.1^+^Ly5.2^+^) cells were isolated from the spleens of each BM chimera as shown in Fig 5 and transferred into Thy1.2^+^Ly5.2^+^ congenic C57BL/6 mice. One day after transfer, the mice were infected with LCMV CL–13. **(B)** Lymphocytes were isolated from the spleens of recipient mice at 5 DPI and analyzed by flow cytometry. Numbers in the plot indicates the frequency of transferred T-E or T-T P14 cells among CD8^+^ T cells in the spleen and absolute numbers of Thy1.1^+^Ly5.1^+^ and Thy1.1^+^Ly5.2^+^ cells in the spleen are summarized in the graph. **(C)** Splenocytes of T-E and T-T P14 chimeric mice were restimulated *in vitro* with GP33 for P14 cells, and frequency of IFN-γ- and TNF-α-producing P14 cells were analyzed. Numbers in the plots indicate the percentage of TNF-α^+^ and TNF-α^-^ P14 cells producing IFN-γ, respectively. Frequency of P14 cells producing both IFN-γ and TNF-α was summarized in the graph. Bar graphs show mean + SD. n≥4 per group in each experiment. **P<0.01; ***P<0.001.

Our observation that IL-4-induced Eomes^+^ innate CD8^+^ T cells are more effective at controlling the CL–13 infection than Eomes^-^ conventional CD8^+^ T cells (Fig 6) raises a question regarding the underlying mechanism. As shown in Fig 5D, Eomes^+^ P14 cells were able to produce more effector cytokine per cell level upon antigen stimulation than Eomes^-^ P14 cells. In addition to an elevated effector function, Eomes^+^ P14 cells also showed better proliferative capability (Fig 6B), resulting in the higher frequency of these cells in the mice that received Eomes^+^ P14 than Eomes^-^ P14 cells (Fig 6C). Therefore, an enhanced quantity and quality of Eomes^+^ P14 cells compared to Eomes^-^ P14 cells could contribute to the accelerated control of CL–13 infection. However, it is possible that functional and numerical differences in the two populations depend on viral titers. To address this issue, we co-transferred both Eomes^+^ and Eomes^-^ P14 cells into same mice (Fig 7A). At 5 DPI, we found that the frequency of Eomes^+^ P14 cells was significantly higher than that of Eomes^-^ P14 cells (Fig 7B). In addition, the ability to produce effector cytokines, IFN-γ and TNF-α, was better in Eomes^+^ P14 cells than in Eomes^-^ P14 cells (Fig 7C). These data indicate that functionality and proliferative capability of Eomes^+^ and Eomes^-^ P14 cells upon antigen stimulation is intrinsically different independently of viral titer.

### Lack of IL-4-induced innate CD8^+^ T cells in CD1d^KO^ BALB/c mice is associated with susceptibility to chronic viral infection

**Fig 8:**
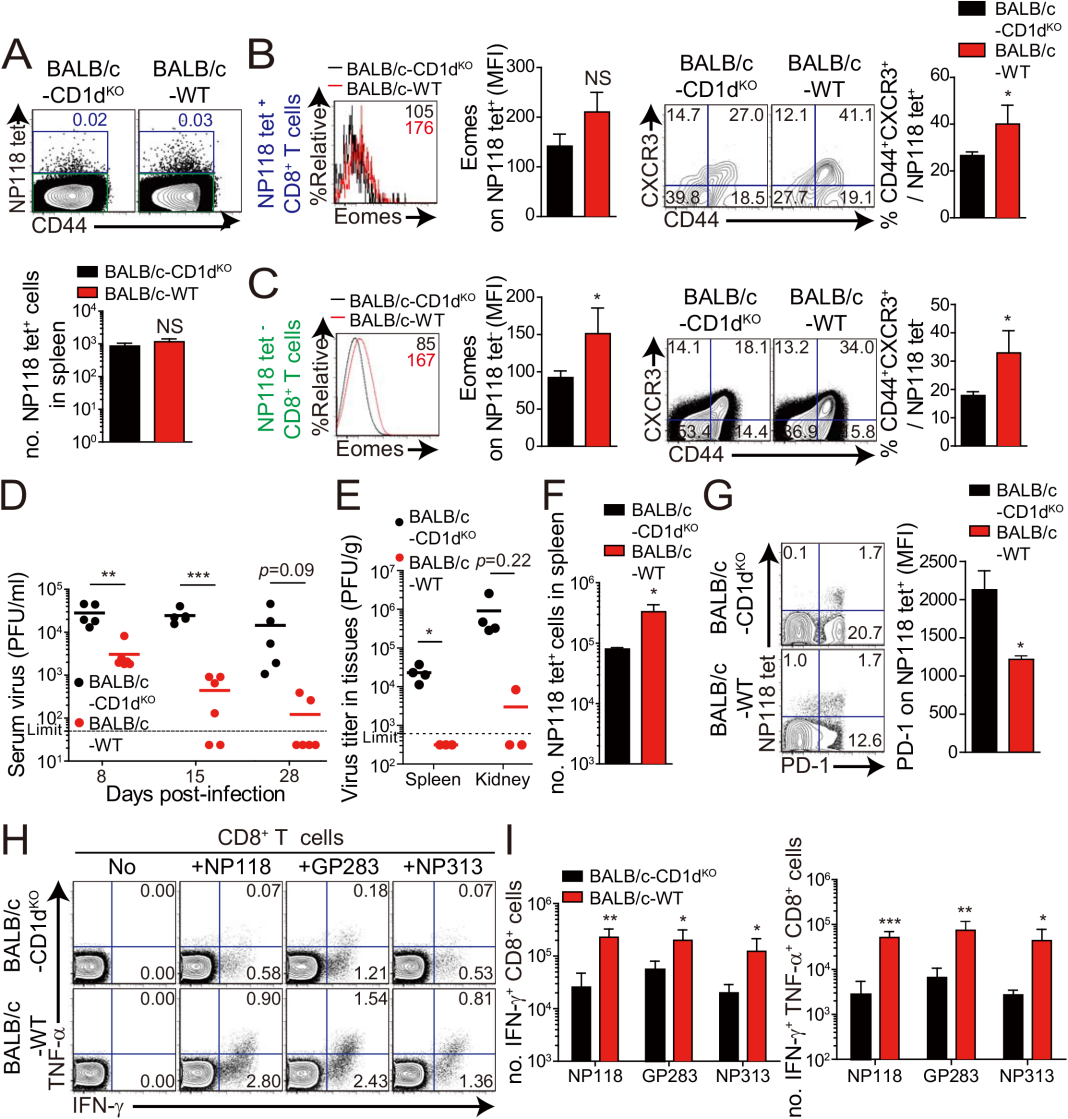
Accelerated viral control in BALB/c wild-type mice compared to BALB/c-CD1d^KO^ mice. (**A-C**) CD8^+^ T cells were enriched from the spleens of BALB/c-CD1d^KO^ and BALB/c wild-type by using magnetic sorting. NP118 tetramer^+^ or tetramer^-^ CD8^+^ T cells were analyzed by flow cytometry. (**A**) NP118 tetramer^+^ cells are gated in blue and NP118 tetramer^-^ cells are gated in green. The numbers in the plots indicated the frequency of NP118 tetramer^+^ cells among CD8^+^ T cells. Absolute number of NP118 tetramer^+^ cells in the spleen is represented in the bar graph. (**B and C**) Eomes expression on NP118 tetramer^+^ or tetramer^-^ cells in BALB/c-CD1d^KO^ and BALB/c wild-type. Numbers in the histogram plots indicate MFI values of Eomes stained on NP118 tetramer^+^ or tetramer^-^ cells. MFI value is summarized in the bar graph. Numbers in the contour plots indicate the frequency of each population in the quadrants depending on CD44 and CXCR3 expression. The frequency of CD44^hi^CXCR3^+^ cells are summarized in the bar graph. (**D-I**) BALB/c-CD1d^KO^ and BALB/c wild-type mice were infected with 2 x 10^5^ PFU per mouse of LCMV CL–13. (**D and E**) Virus titers were checked in serum at the indicated DPI and in spleen and kidney extracted at 29 DPI. Dashed line indicates the virus detection limit. Undetectable samples were given a half of detection limit. (**F**) Absolute numbers of NP118 tetramer-positive CD8^+^ T cells in the spleen and lung of CL–13 infected mice were analyzed at 29 DPI. **(G)** Numbers in plots indicate percentage of PD–1^+^ or PD–1^-^ CD8^+^ T cells. PD–1 expression by NP118 tetramer-positive cells after gating on CD8^+^ T cells were also analyzed, and summary showing the PD–1 expression level on NP118 tetramer-positive CD8^+^ T cells is represented by mean fluorescence intensity (MFI). (**H and I**) Splenocytes were restimulated in vitro with NP118, GP_283-291_ (GP283), or NP_313-322_ (NP313), and frequency of IFN-γ- and TNF-α-producing CD8^+^ T cells were analyzed by flow cytometry (**H**). Numbers in the plots indicate the percentage of TNF-α^+^ and TNF-α^-^ CD8^+^ T cells producing IFN-γ, respectively. Absolute numbers of CD8^+^ T cells producing IFN-γ or both IFN-γ and TNF-α in the spleen are summarized (**I**). Bar graphs show mean + SD. Data are representative of two independent experiments (n≥3 per group in each experiment). *P<0.05; **P<0.01; ***P<0.001.

Next, we wanted to determine whether the anti-viral effect of IL-4-induced innate CD8^+^ T cells also occur in a wild-type host. Unlike C57BL/6 mice, BALB/c mice have an abundant population of IL-4-induced innate CD8^+^ T cells, whose generation is supported by IL–4 produced by intrathymic PLZF^+^ NKT cells [6]. Prior to investigating anti-viral CTL responses in BALB/c strain, we compared IL–4 induced innate CD8^+^ T-cell phenotypes on CD8^+^ T cells in uninfected wild-type BALB/c and CD1d^KO^ BALB/c, in which IL-4-induced innate CD8^+^ T-cell generation is defective due to the absence of NKT cells. The number of virus-specific CD8^+^ T cells, NP_118-126_ (NP118) tetramer^+^ CD8^+^ T cells, was not different between wild-type and CD1d^KO^ BALB/c mice (Fig 8A). However, the NP118 tetramer^+^ CD8^+^ T cells displayed a slightly higher Eomes expression level and the population co-expressing CD44 and CXCR3 among the NP118 tetramer^+^ CD8^+^ T cells was significantly increased in wild-type BALB/c mice than in CD1d^KO^ BALB/c mice (Fig 8B). Similarly, the other CD8^+^ T cells than NP118 tetramer^+^ CD8^+^ T cells also contained the higher population of CD44^hi^CXCR3^+^ cells in wild-type BALB/c mice than in CD1d^KO^ BALB/c mice (Fig 8C), indicating that the frequency of pre-existing innate CD8^+^ T cells is higher in wild-type BALB/c than in CD1d^KO^ BALB/c prior to infection, irrespectively of antigen-specificity of CD8^+^ T cells.

Next, to compare anti-viral CD8^+^ T-cell responses in wild-type BALB/c mice with those of CD1d^KO^ BALB/c mice, we challenged wild-type BALB/c mice and CD1d^KO^ BALB/c mice with CL–13 (2 x 10^5^ PFU/mouse). The viral load in peripheral blood of wild-type BALB/c mice was significantly decreased compared with that of CD1d^KO^ hosts **(** Fig 8D **)**. Moreover, wild-type BALB/c mice exhibited decreased viral titer in spleen and kidney and more abundant virus-specific CD8^+^ T cells in spleen and lungs than CD1d^KO^ mice **(** Fig 8E and 8F **)**. As was the case in CIITA^Tg^ mice, PD–1 expression on virus-specific CD8^+^ T cells in wild-type BALB/c mice was not sustained at a later time point after viral infection in wild-type BALB/c mice (Fig 8G). Furthermore, we observed that CD8^+^ T cells from wild-type BALB/c mice produced higher amounts of effector cytokines such as IFN-γ and TNF-α than did those from CD1d^KO^ mice **(** Fig 8H and 8I **)**. These data suggest that IL-4-induced innate CD8^+^ T cells in a wild-type BALB/c host also contributed to reduce CL–13 viral load compared with CD1d^KO^ mice.

## Discussion

Over the course of viral infection there may be a limited time period during which the host system can eliminate the virus [22]. When viruses are not eliminated within this period of time, virus-specific CD8^+^ T cells are exhausted via PD–1 (programmed death 1) and its ligand, PD-L1 interaction, resulting in a chronic infection [23]. In this study we demonstrated that IL-4-induced innate CD8^+^ T cells are able to effectively control the chronic viral infection. For this, we first compared T-cell responses to chronic viral infection induced by LCMV CL–13 in CIITA^Tg^ and wild-type C57BL/6 mice. The immune system of the CIITA^Tg^ mouse resembles that of humans with respect to MHC class II expression in both thymic epithelial cells and thymocytes making it a suitable model [24–27]. Thus, PLZF^+^ T-T CD4^+^ T cells are generated in response to TCR signals from the MHC class II/peptide complex expressed on thymocytes [15,27,28] and provide IL–4 for the development of IL-4-induced innate CD8^+^ T cells [14]. When mice were infected with CL–13, CIITA^Tg^ mice were able to control viral titers below detection levels in selected tissues such as the spleen and serum within a month, whereas wild-type mice succumbed to persistent infection. Furthermore, the ability of CIITA^Tg^ mice to control the virus was dependent on IL-4-induced innate CD8^+^ T cells as CIITA^Tg^ and control mice did not show a difference in serum viral titers on the IL–4 deficient background, which causes a lack of intrathymic generation of IL-4-induced innate CD8^+^ T cells. Moreover, adoptive transfer of LCMV-specific IL-4-induced innate CD8^+^ T cells into wild-type hosts further confirmed the crucial role of these innate T cells as the primary effector mechanism for viral control. We also demonstrated that wild-type BALB/c mice, which have abundant IL-4-induced innate CD8^+^ T cells, exhibit notably enhanced anti-viral CTL responses compared with CD1d^KO^ BALB/c mice, which only possess a very small fraction of these cells. As expected, expression level of Eomes and innate CD8^+^ T cell marker (CD44 and CXCR3) was higher in virus-specific CD8^+^ T cells wild-type mice, as compared to those of CD1d^KO^ BALB/c mouse (Fig 8B), while we could not found any difference in LCMV NP118-specific CD8^+^ T cell numbers in these mice (Fig 8A). Considering the results from P14 cell adoptive transfer and CL–13 infection (Fig 6), these data favor the idea that IL-4-induced innate CD8^+^ T cells in a wild-type BALB/c host contributed to reduce CL–13 viral load compared with CD1d^KO^ mice, although the genetic perturbation in CD1d^KO^ mice is not specific for the innate CD8^+^ T cell population.

Eomes is a key transcription factor in the cytotoxic T-cell lineage [29]. During the activation and differentiation of mature CD8^+^ T cells Eomes induces effector function and cooperates with T-bet to sustain memory CD8^+^ T-cell homeostasis [30]. In particular, the central memory population is diminished in CD8^+^ T cells lacking Eomes [29,30]. Moreover, in the thymus Eomes also seems to confer effector function and memory phenotypes to innate CD8^+^ T cells as IL-4-induced innate CD8^+^ T cells acquire a CD44^hi^CD62L^hi^ central memory cell-like phenotype [14]. In addition to the previous reports, our comparison data of phenotype and function in between IL-4-induced innate CD8^+^ T cells and memory CD8^+^ T cells showed that the expression levels of Eomes and CXCR3 were similar but those of CD44, CD124, CD24, and NKG2D were different (S3A Fig). IFN-γ production and degranulation ability upon antigen stimulation were also different (S3B Fig). Although these IL-4-induced innate CD8^+^ T cells are less functional than memory CD8^+^ T cells, their function is evidently better than those of naïve CD8^+^ T cells. These data demonstrate that IL-4-induced innate CD8^+^ T cells are phenotypically and functionally different from conventional memory CD8^+^ T cells as well as naïve CD8^+^ T cells.

On the other hand, the expression of Eomes mRNA and protein are markedly elevated in exhausted CD8^+^ T cells during chronic virus infection compared to that in effector or memory CD8^+^ T cells [20,31]. These finding suggest that Eomes alone is not sufficient to stimulate the effector function of exhausted CD8^+^ T cells under the conditions of established chronic virus infection. This is despite the fact that upregulation of Eomes initially triggers the effector function of CD8^+^ T cells upon TCR stimulation and contributes to preserve the functionality of memory CD8^+^ T cells. In the present study, when CD8^+^ T cells already express a significantly high level of Eomes, these cells acquire obviously enhanced ability to produce effector cytokines such as IFN-γ and TNF-α upon viral antigen challenge. Taken together, these data suggest that a high level of Eomes expression allows IL-4-induced innate CD8^+^ T cells to exhibit their prompt and significant effector function, thereby controlling viremia during the early phase of virus infection.

Many researchers have attempted to control chronic virus infection using immunotherapeutic interventions such as blockade of the inhibitory receptors PD–1 and CTLA–4, administration of type I IFN, and regulation of microRNAs [32] [33,34]. Additionally, for chronic hepatitis B virus infection treatment, adoptive T-cell therapy using either *in vitro* expanded hepatitis B virus antigen-specific T cells or grafting T cells with recombinant TCR has been investigated as an approach [35]. Based on our data, virus-specific IL-4-induced innate CD8^+^ T cells have the potential to be used in adoptive T-cell therapy. Interestingly, when we adoptively transferred Eomes^+^ LCMV-specific innate CD8^+^ T cells into CL-13-infected mice, the CTL response was slightly increased even though the transferred cells were seemed still exhausted (S4 Fig). From this experiment, we hypothesize that combination therapy with adoptive transfer of IL-4-induced innate CD8^+^ T cells for prompt control of virus and PD–1 blockade for rejuvenating CTL function could be effective. Further experiments will be required to test this theory.

X-linked lymph proliferative disease (XLP) is a human immunodeficiency caused by germ-line mutations in *SH2D1A* gene and characterized by an inability to respond appropriately to infections such as Epstein-Barr virus [36]. The *SH2D1A* gene encodes the SAP molecule that is a component of the SLAM (signaling lymphocytic activation molecule) signaling pathway. Signaling through the SLAM family of receptors is crucial for the development of NKT cells, and thus the absence of the SAP causes an arrest in NKT cell development [37] [38,39]). In this context, the deficiency of NKT cells has been considered as one of the cellular bases of XLP [38]. However, considering the crucial role of SAP for T-T CD4^+^ T-cell development [40], the development and function of T-T CD4^+^ T cells would also be expected to be defective in XLP patients [27]. Both NKT and T-T CD4^+^ T cells are engaged in the generation of innate CD8^+^ T cells via IL–4 production and thus, the development of IL-4-induced innate CD8^+^ T cells is also dependent on the adaptor SAP [41]. In the present study, we demonstrated that IL-4-induced innate CD8^+^ T cells are able to rapidly proliferate, secrete cytokines, and decrease viral load after LCMV CL–13 infection. Taken together, it is necessary to consider the possibility that defective development of IL-4-induced innate CD8^+^ T cells causes the heighten susceptibility to Epstein-Barr virus infection in XLP patients.

## Materials and Methods

### Ethics statement

Animals were maintained and procedures were performed with approval of the IACUCs of Seoul National University (permit number: SNUIBC-R100524-1) and Yonsei University (permit number: 2013–0115) in accordance to LABORATORY ANIMAL ACT of Korean Ministry of Food and Drug Safety for enhancing the ethics and reliability on animal testing through appropriate administration of laboratory animals and animal testing.

### Mice, infection, and titration

C57BL/6, IL-4^KO^, BALB/c and BALB/c-CD1d^KO^ mice were purchased from the Jackson Laboratory. The CIITA^Tg^ mice were previously generated at Seoul National University [26] and CIITA^Tg^ mice were bred to IL-4^KO^ and PIV^KO^ mice in our laboratory to generate CIITA^Tg^IL-4^KO^ and CIITA^Tg^PIV^KO^. LCMV epitope-specific TCR transgenic P14 Thy1.1^+^ Ly5.2^+^ mice were obtained from the Emory Vaccine Center, USA and P14 Thy1.1^+^ Ly5.1^+^ mice were obtained from POSTECH, Korea. All mice were maintained in the specific pathogen-free facility of the Yonsei Laboratory Animal Research Center at Yonsei University and the Center for Animal Resource Development at Seoul National University College of Medicine (Seoul, Korea). LCMV CL–13, a variant derived from an LCMV ARM CA1371 carrier mouse [42], was obtained from Rafi Ahmed (Emory Vaccine Center, Atlanta). Six- to ten-week old mice were infected with 1 x 10^5^ to 2 x 10^6^ PFU of LCMV CL–13 diluted in serum-free RPMI medium per 20 g of mouse body weight by intravenous infection or with 2 x 10^5^ PFU of LCMV Armstrong diluted in serum-free RPMI medium per 20 g of mouse body weight by intraperitoneal infection. For serum virus titration, three to four drops of blood were individually collected by microcapillary tube at the indicated time points post infection, and the serum was directly stored at -70°C. For tissue titration, small pieces of the spleen and kidney were put in DMEM containing 1% FBS (HyClone) and stored at -70°C. The tissues were later homogenized completely using a homogenizer (Kinematica) before titration. Viral titers from sera or homogenized samples were determined by plaque assay on Vero cells as previously described [43]. Undetectable samples were given a half of each detection limit.

### Cell isolation, antibodies, and staining

Peripheral blood mononuclear cells (PBMCs) were isolated from peripheral blood using density gradient centrifugation underlaid with Histopaque–1077 (Sigma-Aldrich). Lymphocytes from the thymus, spleen and lung were isolated as previously described [23]. For phenotypic analysis of lymphocytes, single-cell suspensions were stained with the following antibodies; fluorochrome-conjugated antibodies against CD4 (RM4-5), CD127 (A7R34), and PD–1 (RMP1-13) were from BioLegend, antibodies against CD8 (53–6.7), CD24 (30-F1), CD44 (IM7), CXCR3 (CXCR3-173), and CD19 (eBio1D3) were from eBioscience, and antibodies against CD44 (IM7), CD90.1 (Thy1.1; OX–1), CD122 (IL-2Rβ; TM-b1), CD124 (IL-4Rα; mIL4R-M1), CXCR5 (2G8), CD45R/B220 (RA3-6B2), CD95 (Fas; Jo2), T- and B-cell activation antigen (GL7), and NKG2D (CX5) were from BD Biosciences. H-2D^b^ tetramers bound to GP_33-41_ or GP_276-286_ peptide and H-2L^d^ tetramer bound to NP_118-126_ peptide were generated and used as previously described [44]. To detect cytokine production by virus-specific CD8^+^ T cells, splenocytes from C57BL/6 mice were restimulated *in vitro* with 0.2 μg/ml of LCMV GP_33-41_, GP_276-286_, or peptide pool including GP_33-41_, GP_276-286_, GP_70-77_, GP_92-101_, NP_166-175_, NP_205-212_, NP_235-249_, and NP_396-404_ in the presence of Golgi plug/Golgi stop (BD Biosciences) and anti-CD107a (1D4B, BD Biosciences) Ab for 5 hours followed by intracellular cytokine staining using anti-IFN-γ (XMG1.2, BD Biosciences) and anti-TNF-α (MP6-XT22, BioLegend) antibodies. In case of *in vitro* restimulation of splenocytes from BALB/c mice, LCMV NP_118-126_, GP_283-291_, or NP_313-322_ peptide was used. For IL-4-induced innate CD8^+^ T-cell staining, lymphocytes were fixed and permeabilized with Fixation/Permeabilization solution (eBioscience) and stained with anti-Eomes (Dan11mag, eBioscience) Ab. The Live/Dead fixable Stain Kit (Invitrogen) was used to remove the dead cell population in most staining procedures. All stained samples were read by FACS CANTO II or LSR II (BD Biosciences), and analyzed by FlowJo software (Tree Star).

### Histopathology

Lungs were fixed in 10% neutral buffered formalin (Sigma-Aldrich), and paraffin-embedded tissues were sectioned to a thickness of 4 μm and stained with hematoxylin and eosin. Microscopic observations were performed with an ECLIPSE 80i Bright-Field Microscope Set (Nikon) equipped with CFI 10×/22 eyepiece, Plan Fluor objectives (with 4×, 10×, 20×, and 100× objectives) and DS-Fi1 camera. We used NIS-Elements BR 3.1 software (Nikon) for image acquisition.

### ELISA (enzyme-linked immunosorbent assay)

LCMV-specific antibodies were measured by ELISA. LCMV CL-13-infected BHK–21 cell lysate was used as capture antigen. Ninety-six-well Polysorp plates (Nunc) were coated with sonicated lysate for 3 days before performing the ELISA. Three fold serial dilutions of serum samples were incubated and detected with IgG-specific horseradish peroxidase (HRP)-conjugated goat anti-mouse immunoglobulin (Southern Biotech). TMB (Sigma-Aldrich) was used as a substrate, and the reaction was stopped by sulfuric acid and read at 450nm.

### BM chimeras

BM cells were isolated from femurs and tibias of donor mice and T cells were depleted through magnetic sorting using a mixture of CD4 and CD8 depleting microbeads (Miltenyi Biotech, Auburn, CA). Then, 3 x 10^6^ T cell-depleted BM cells were intravenous injected into each recipient mouse preconditioned with 13,000 rad irradiation (two doses of 650 rad applied 4 h apart) from a ^137^Cs source 1 day prior.

### Cell purification and adoptive transfer

For CD8^+^ T cell enrichment, CD8^+^ T cells were isolated from the spleen of wild-type BALB/c and CD1d^KO^ BALB/c mice after negative selection using a CD8^+^ T-cell isolation kit (Miltenyi Biotech).

For adoptive transfer of P14 Thy1.1^+^ Ly5.2^+^ CD8^+^ T cells and P14 Thy1.1^+^ Ly5.1^+^ CD8^+^ T cells, the cells were isolated from the spleen of BMT chimera mice after negative selection using a CD8^+^ T-cell isolation kit and subsequent positive selection using Thy1.1^+^ cell microbeads (Miltenyi Biotech). After isolation, 5 x 10^3^ purified P14 Thy1.1^+^ CD8^+^ T cells were transferred alone or with P14 Ly5.1^+^ CD8^+^ T cells into wild-type C57BL/6 mice via tail vein. Mice were infected with LCMV CL–13 at 1d after the adoptive transfer.

### In vivo proliferation assay

LCMV GP_33-41_-specific P14 CD8^+^ T cells were isolated from P14 transgenic mice using CD8^+^ isolation kit (Myltenyi Biotec). Purified P14 CD8^+^ T cells were labelled with CellTrace Violet (CTV) proliferation kit at concentration of 10uM (Invitrogen). Labelled P14 CD8^+^ T cells (1 x 10^6^ cells) were adoptively transferred into naïve mice. Mice were infected with LCMV CL–13 at 1d after the adoptive transfer.

### Therapeutic adoptive transfer

Thy1.2^+^ congenic C57BL/6 mice were infected with 2 x 10^6^ PFU of LCMV CL–13. At 3 weeks post infection, P14 Thy1.1^+^ CD8^+^ T cells were isolated from the spleen of BMT chimera mice for adoptive transfer after negative selection using a CD8^+^ T-cell isolation kit and subsequent positive selection using Thy1.1^+^ cell microbeads (Miltenyi Biotech). After isolation, 2 x 10^6^ purified P14 Thy1.1^+^ CD8^+^ T cells were transferred into CL-13-infected C57BL/6 mice via tail vein.

### Statistical analysis

Survival curve was evaluated by log-rank (Mantel-Cox) test and other data were analyzed by two-tailed unpaired Student’s *t*-test using GraphPad Prism software. A *p* value less than 0.05 was considered statistically significant.

## Supporting Information

S1 Fig The minimal infectious dose allowing chronic infection in wild-type C57BL/6 mice. Wild-type B6 mice were infected with the indicated dose of LCMV CL–13. PBMCs (A) and serum (B) were collected at indicated DPI. The PD–1 expression level on GP33 tetramer-positive CD8^+^ T cells was analyzed by flow cytometry, and summarized data are represented by mean fluorescence intensity (MFI) value (A). Serum viral titer was also measured in each group of mice at the indicated DPI (B). Dashed line indicates the virus detection limit. Undetectable samples were given a half of detection limit. Bar graphs show mean + SD. Data are representative of at least two independent experiments (n≥3 per group in each experiment). NS, not significant.
(PDF)

S2 Fig No effects on CD8^+^ T-cell responses and antibody responses upon LCMV CL–13 infection by IL–4 deficiency. Wild-type and IL-4^KO^ mice were infected with 2 x 10^6^ PFU per mouse of LCMV CL–13. PBMCs (A and B) and serum (C and F) were collected at indicated DPI. The numbers of GP33 tetramer-positive CD8^+^ T cells per 10^6^ PBMCs during the course of LCMV CL–13 infection are represented in (A). (B) PD–1 expression level on GP33 tetramer-positive cells were summarized by MFI. (C) Kinetics of LCMV-specific IgG was detected by ELISA. (D and E) Lymphocytes were isolated from the spleen of LCMV CL-13-infected wild-type and IL-4^KO^ mice at 33 DPI and analyzed by flow cytometry. (D) Absolute numbers of CD4^+^ CXCR5^+^ PD–1^+^ T_FH_ cells in the spleen are represented. (E) Absolute numbers of CD19^+^ B220^+^ Fas^+^ GL7^+^ GC B cells in the spleen are also summarized in bar graph. Viral titers in serum (F) and in the spleen extracted from LCMV CL-13-infected mice at 33 DPI (G) were checked. Dashed line indicates the virus detection limit. Undetectable samples were given a half of detection limit. Line graph shows mean ± SD. Bar graphs show mean + SD. Data are representative of three independent experiments (n≥3 per group in each experiment). NS, not significant; *P<0.05.
(PDF)

S3 Fig The phenotypic and functional differences between innate CD8^+^ T cells and conventional memory CD8^+^ T cells. T-T P14 cells were isolated from the spleens of each BM chimera as shown in Fig 5A. Naïve P14 cells were isolated from the spleens of P14 Thy1.1^+^ transgenic mice and transferred into C57BL/6 wild-type mice. One day after transfer, the mice were infected with 2 x 10^5^ PFU per mouse of LCMV Armstrong (Arm). Memory P14 cells were analyzed from the spleen of the infected mice at 90 DPI. (A) Eomes, CD44, CXCR3, CD124, CD24, and NKG2D expression levels were compared on T-T P14 with memory P14 cells, and summarized in the graphs. Naïve P14 cells were used as control. (B) Splenocytes from the T-T P14 BM chimeric mice and LCMV Arm-infected mice were stimulated *in vitro* with GP33 for P14 cells, and stained with CD107a and IFN-γ. Numbers in quadrants indicate the percentage of CD107a^+^ IFN-γ-producing or nonproducing P14 cells. n = 1 per group for naïve group and n≥3 per group for T-T and memory group in this experiment. NS, not significant; *P<0.05; **P<0.01.
(PDF)

S4 Fig Therapeutic adoptive transfer of Eomes^+^ LCMV-specific innate CD8^+^ T cells into CL-13-infected mice. (A) Thy1.2^+^ congenic C57BL/6 wild-type mice were infected with 2 x 10^6^ PFU per mouse of LCMV CL–13, followed by adoptive transfer of T-E or T-T P14 cells (Thy1.1^+^) into the infected mice at 3 weeks post infection. Four weeks after adoptive transfer, lymphocytes were isolated from the spleen of the infected mice and analyzed by flow cytometry. (B) The number of Thy1.1^+^ P14 cells and their PD–1 expression were analyzed. Numbers in the plots indicate PD–1^+^ or PD–1^-^ Thy1.1^+^ transferred cells. PD–1 expression level (MFI) on Thy1.1^+^ transferred cells is summarized in the graph. (C and D) Splenocytes of CL–13 infected mice that received T-E or T-T P14 cells were restimulated *in vitro* with GP33, GP276, and peptide pool. (C) The frequency of IFN-γ- and TNF-α-producing Thy1.1^+^ transferred cells was analyzed. Numbers in the plots indicate the percentage of TNF-α^+^ and TNF-α^-^ CD8^+^ T cells producing IFN-γ, respectively. Frequency of Thy1.1^+^ transferred cells producing both IFN-γ and TNF-α was summarized in the graph. (D) The frequency of IFN-γ- and TNF-α-producing CD8^+^ T cells was analyzed by flow cytometry. Numbers in the plots indicate the percentage of TNF-α^+^ and TNF-α^-^ CD8^+^ T cells producing IFN-γ, respectively. Absolute numbers of CD8^+^ T cells producing IFN-γ in the spleen were summarized in the bar graph. n = 5 per group in the experiment. NS, not significant; *P<0.05.
(PDF)
