# Supplementary figures and images for: IL-4 Induced Innate CD8+ T Cells Control Persistent Viral Infection
Source: PLoS Pathog. 2015 Oct 9;11(10):e1005193. doi: 10.1371/journal.ppat.1005193 (PMC4599894; doi:10.1371/journal.ppat.1005193)

**A**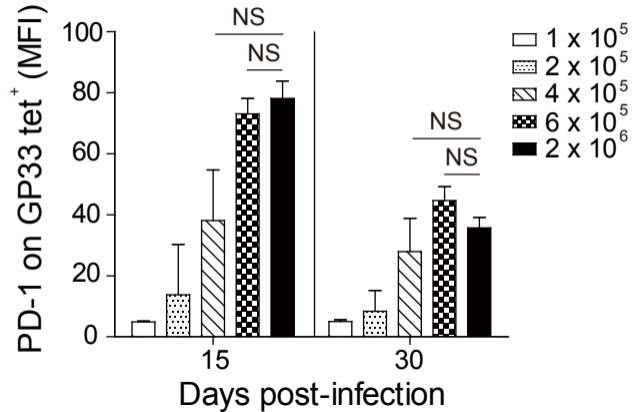**B**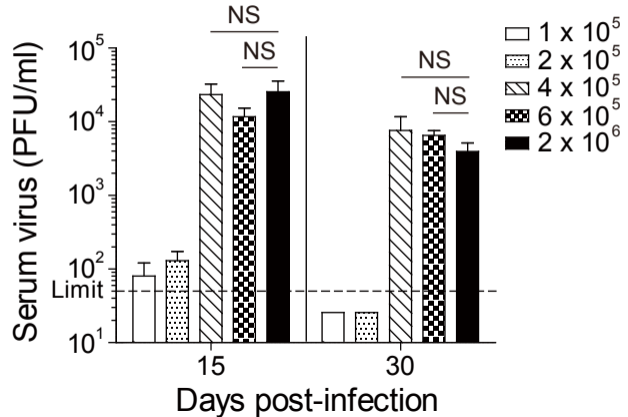

Supplement: S1 Fig — Wild-type B6 mice were infected with the indicated dose of LCMV CL–13. PBMCs (A) and serum (B) were collected at indicated DPI. The PD–1 expression level on GP33 tetramer-positive CD8+ T cells was analyzed by flow cytometry, and summarized data are represented by mean fluorescence intensity (MFI) value (A). Serum viral titer was also measured in each group of mice at the indicated DPI (B). Dashed line indicates the virus detection limit. Undetectable samples were given a half of detection limit. Bar graphs show mean + SD. Data are representative of at least two independent experiments (n≥3 per group in each experiment). NS, not significant. (PDF) [file ppat.1005193.s001.pdf]

**A**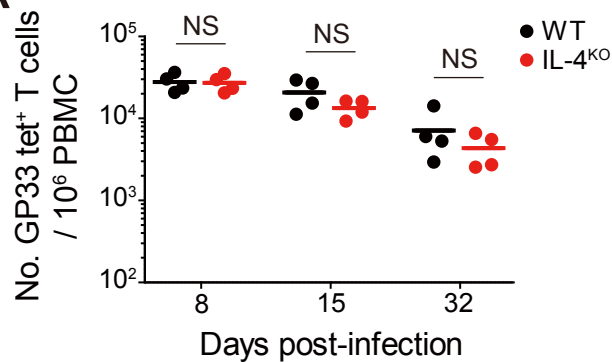**B**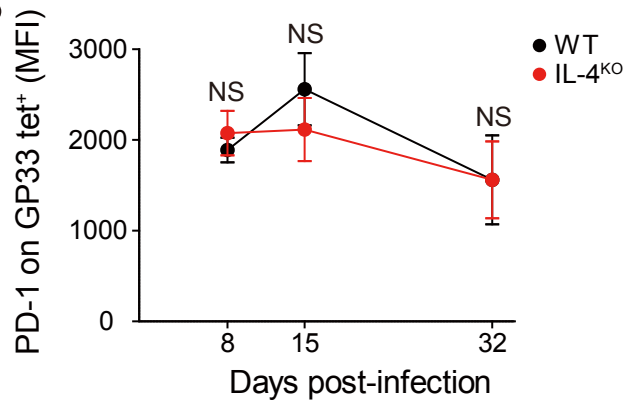**C**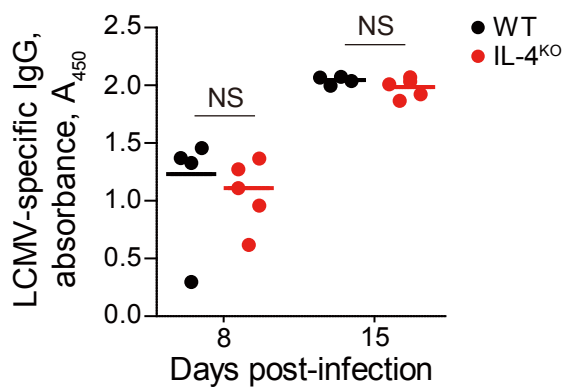**D**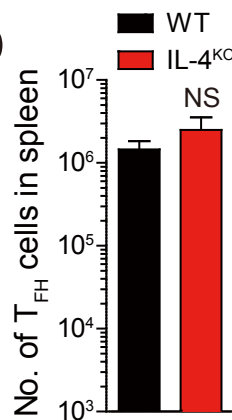**E**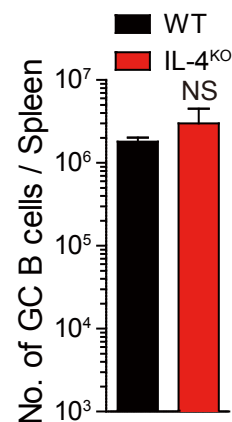**F**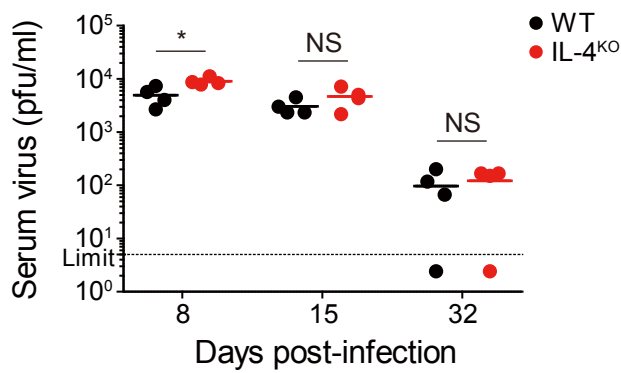**G**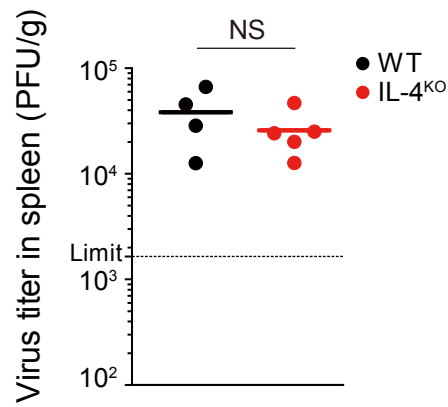

Supplement: S2 Fig — Wild-type and IL-4KO mice were infected with 2 x 10^6^ PFU per mouse of LCMV CL–13. PBMCs (A and B) and serum (C and F) were collected at indicated DPI. The numbers of GP33 tetramer-positive CD8+ T cells per 106 PBMCs during the course of LCMV CL–13 infection are represented in (A). (B) PD–1 expression level on GP33 tetramer-positive cells were summarized by MFI. (C) Kinetics of LCMV-specific IgG was detected by ELISA. (D and E) Lymphocytes were isolated from the spleen of LCMV CL-13-infected wild-type and IL-4KO mice at 33 DPI and analyzed by flow cytometry. (D) Absolute numbers of CD4+ CXCR5+ PD–1+ TFH cells in the spleen are represented. (E) Absolute numbers of CD19+ B220+ Fas+ GL7+ GC B cells in the spleen are also summarized in bar graph. Viral titers in serum (F) and in the spleen extracted from LCMV CL-13-infected mice at 33 DPI (G) were checked. Dashed line indicates the virus detection limit. Undetectable samples were given a half of detection limit. Line graph shows mean ± SD. Bar graphs show mean + SD. Data are representative of three independent experiments (n≥3 per group in each experiment). NS, not significant; *P<0.05. (PDF) [file ppat.1005193.s002.pdf]

**A**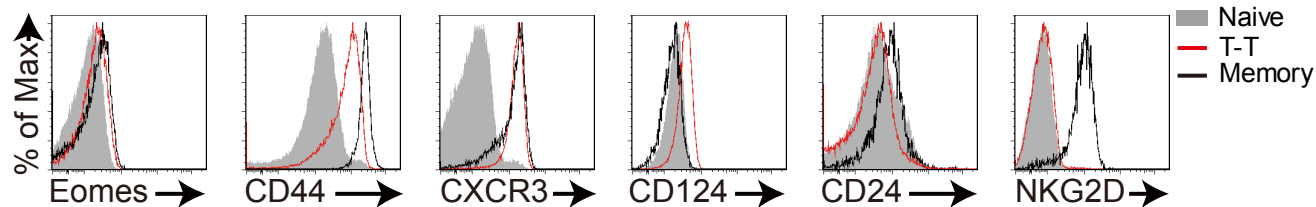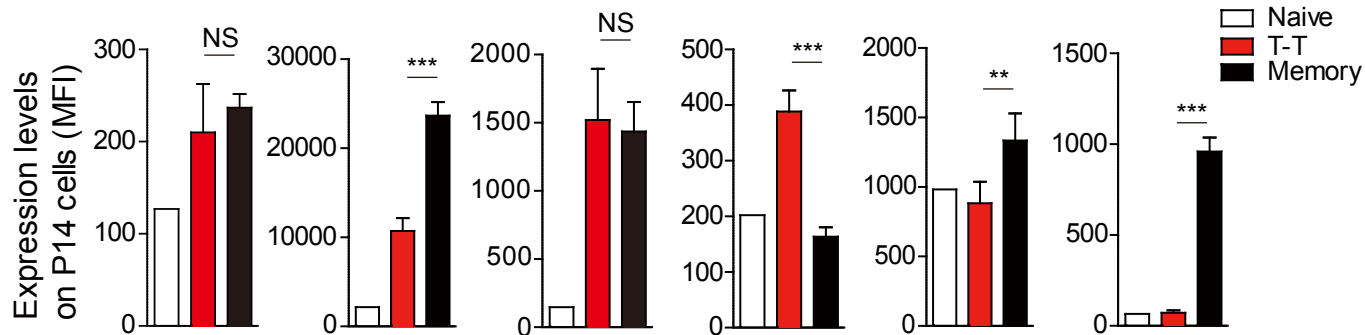**B**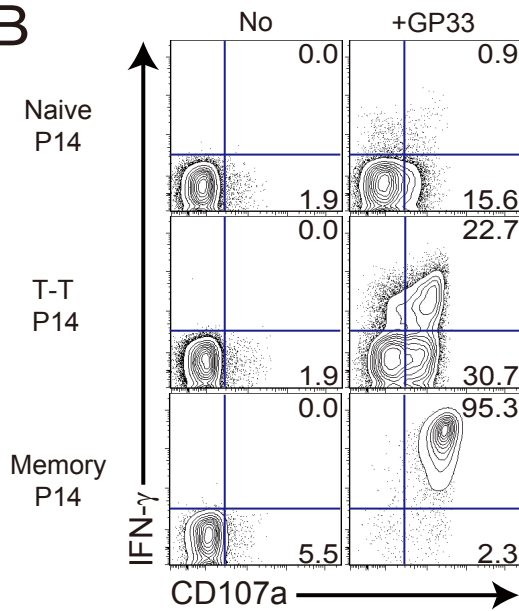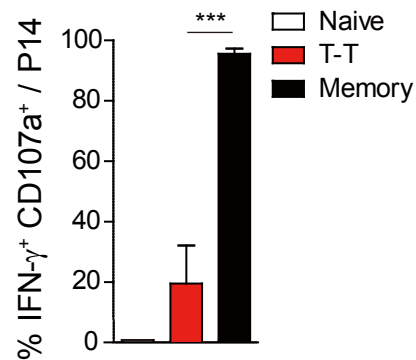

Supplement: S3 Fig — T-T P14 cells were isolated from the spleens of each BM chimera as shown in Fig 5A. Naïve P14 cells were isolated from the spleens of P14 Thy1.1+ transgenic mice and transferred into C57BL/6 wild-type mice. One day after transfer, the mice were infected with 2 x 10^5^ PFU per mouse of LCMV Armstrong (Arm). Memory P14 cells were analyzed from the spleen of the infected mice at 90 DPI. (A) Eomes, CD44, CXCR3, CD124, CD24, and NKG2D expression levels were compared on T-T P14 with memory P14 cells, and summarized in the graphs. Naïve P14 cells were used as control. (B) Splenocytes from the T-T P14 BM chimeric mice and LCMV Arm-infected mice were stimulated in vitro with GP33 for P14 cells, and stained with CD107a and IFN-γ. Numbers in quadrants indicate the percentage of CD107a+ IFN-γ-producing or nonproducing P14 cells. n = 1 per group for naïve group and n≥3 per group for T-T and memory group in this experiment. NS, not significant; *P<0.05; **P<0.01. (PDF) [file ppat.1005193.s003.pdf]

**A**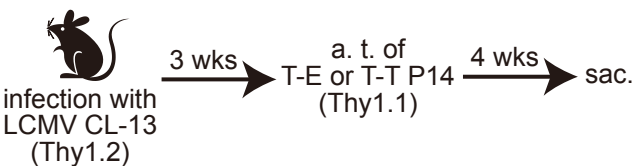**B**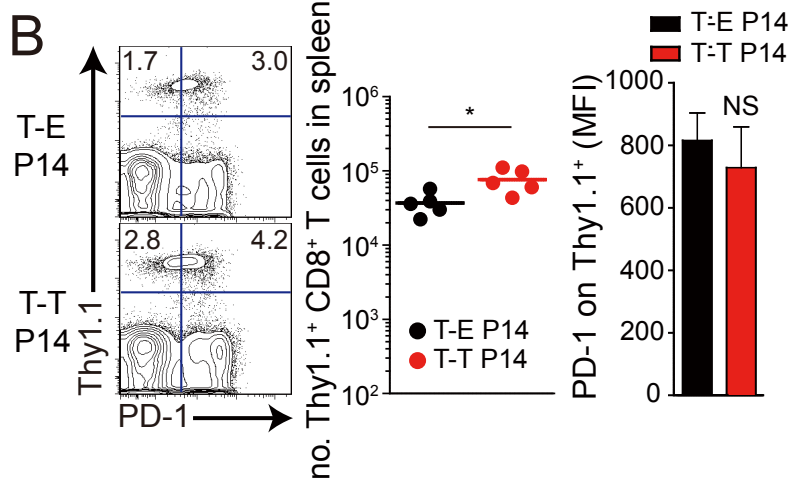**C**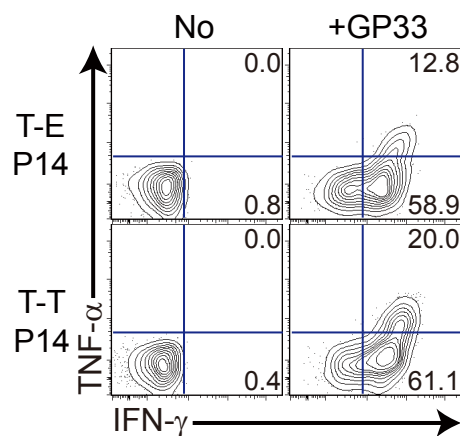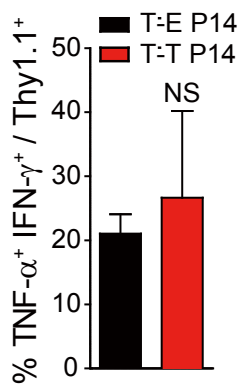**D**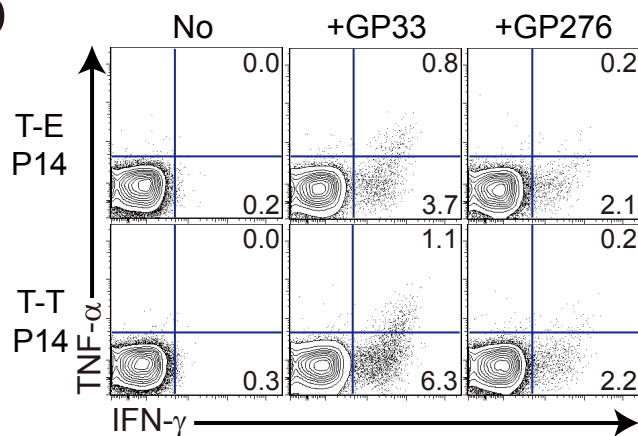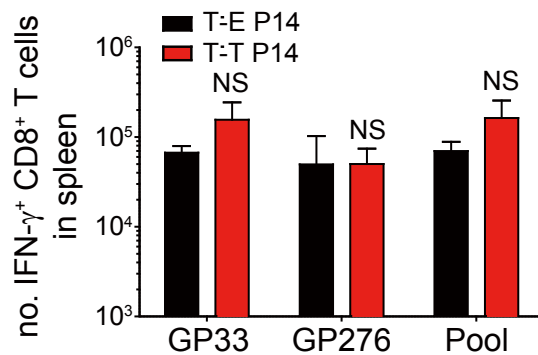

Supplement: S4 Fig — (A) Thy1.2+ congenic C57BL/6 wild-type mice were infected with 2 x 10^6^ PFU per mouse of LCMV CL–13, followed by adoptive transfer of T-E or T-T P14 cells (Thy1.1+) into the infected mice at 3 weeks post infection. Four weeks after adoptive transfer, lymphocytes were isolated from the spleen of the infected mice and analyzed by flow cytometry. (B) The number of Thy1.1+ P14 cells and their PD–1 expression were analyzed. Numbers in the plots indicate PD–1+ or PD–1- Thy1.1+ transferred cells. PD–1 expression level (MFI) on Thy1.1+ transferred cells is summarized in the graph. (C and D) Splenocytes of CL–13 infected mice that received T-E or T-T P14 cells were restimulated in vitro with GP33, GP276, and peptide pool. (C) The frequency of IFN-γ- and TNF-α-producing Thy1.1+ transferred cells was analyzed. Numbers in the plots indicate the percentage of TNF-α+ and TNF-α- CD8+ T cells producing IFN-γ, respectively. Frequency of Thy1.1+ transferred cells producing both IFN-γ and TNF-α was summarized in the graph. (D) The frequency of IFN-γ- and TNF-α-producing CD8+ T cells was analyzed by flow cytometry. Numbers in the plots indicate the percentage of TNF-α+ and TNF-α- CD8+ T cells producing IFN-γ, respectively. Absolute numbers of CD8+ T cells producing IFN-γ in the spleen were summarized in the bar graph. n = 5 per group in the experiment. NS, not significant; *P<0.05. (PDF) [file ppat.1005193.s004.pdf]
